# End-to-end learning of multiple sequence alignments with differentiable Smith–Waterman

**DOI:** 10.1093/bioinformatics/btac724

**Published:** 2022-11-10

**Authors:** Samantha Petti, Nicholas Bhattacharya, Roshan Rao, Justas Dauparas, Neil Thomas, Juannan Zhou, Alexander M Rush, Peter Koo, Sergey Ovchinnikov

**Affiliations:** NSF-Simons Center for the Mathematical and Statistical Analysis of Biology, Harvard University, Cambridge, MA 02138, USA; Department of Mathematics, University of California Berkeley, Berkeley, CA 94720, USA; Electrical Engineering and Computer Sciences, University of California Berkeley, Berkeley, CA 94720, USA; Institute for Protein Design, University of Washington, Seattle, WA 98195, USA; Electrical Engineering and Computer Sciences, University of California Berkeley, Berkeley, CA 94720, USA; Department of Biology, University of Florida, Gainesville, FL 32611, USA; Department of Computer Science, Cornell Tech, New York, NY 10044, USA; Simons Center for Quantitative Biology, Cold Spring Harbor Laboratory, Cold Spring Harbor, NY 11724, USA; John Harvard Distinguished Science Fellowship, Harvard University, Cambridge, MA 02138, USA

## Abstract

**Motivation:**

Multiple sequence alignments (MSAs) of homologous sequences contain information on structural and functional constraints and their evolutionary histories. Despite their importance for many downstream tasks, such as structure prediction, MSA generation is often treated as a separate pre-processing step, without any guidance from the application it will be used for.

**Results:**

Here, we implement a smooth and differentiable version of the Smith–Waterman pairwise alignment algorithm that enables jointly learning an MSA and a downstream machine learning system in an end-to-end fashion. To demonstrate its utility, we introduce SMURF (Smooth Markov Unaligned Random Field), a new method that jointly learns an alignment and the parameters of a Markov Random Field for unsupervised contact prediction. We find that SMURF learns MSAs that mildly improve contact prediction on a diverse set of protein and RNA families. As a proof of concept, we demonstrate that by connecting our differentiable alignment module to AlphaFold2 and maximizing predicted confidence, we can learn MSAs that improve structure predictions over the initial MSAs. Interestingly, the alignments that improve AlphaFold predictions are self-inconsistent and can be viewed as adversarial. This work highlights the potential of differentiable dynamic programming to improve neural network pipelines that rely on an alignment and the potential dangers of optimizing predictions of protein sequences with methods that are not fully understood.

**Availability and implementation:**

Our code and examples are available at: https://github.com/spetti/SMURF.

**Supplementary information:**

Supplementary data are available at *Bioinformatics* online.

## 1 Introduction

Multiple sequence alignments (MSAs) are commonly used in biology to model evolutionary relationships and the structural/functional constraints within families of proteins and RNA. MSAs are a critical component of the latest contact (Balakrishnan *et al.*, 2011; Jones *et al.*, 2012; Morcos *et al.*, 2011) and protein structure prediction pipelines (Baek *et al.*, 2021; Jumper *et al.*, 2021). Moreover, they are used for predicting the functional effects of mutations (Figliuzzi *et al.*, 2016; Frazer *et al.*, 2021; Hopf *et al.*, 2017; Sundaram *et al.*, 2018), phylogenetic inference (Felsenstein and Felenstein, 2004) and rational protein design (Goldenzweig *et al.*, 2016; Ma *et al.*, 2016; Russ *et al.*, 2020; Tian *et al.*, 2018). Creating alignments, however, is a challenging problem. Standard approaches use heuristics for penalizing substitutions and gaps and do not take into account the effects of contextual interactions (Steinegger *et al.*, 2019) or long-range dependencies. For example, these local approaches struggle when aligning large numbers of diverse sequences, and additional measures (such as the introduction of external guide Hidden Markov Models, HMMs) must be introduced to obtain reasonable alignments (Sievers and Higgins, 2014). Finally, each alignment method has a number of hyperparameters which are often chosen on an application-specific basis. This suggests that computational methods that input an MSA could be improved by jointly learning the MSA and training the method.

Here, we introduce the *Learned Alignment Module* (LAM), which is a fully differentiable module for constructing MSAs and hence can be trained in conjunction with another differentiable downstream model. Building upon the generalized framework for differentiable dynamic programming developed in Mensch and Blondel (2018), LAM employs a smooth and differentiable version of the Smith–Waterman algorithm. Whereas the classic implementation of the Smith–Waterman algorithm outputs a pairwise alignment between two sequences that maximizes an alignment score (Smith and Waterman, 1981), the smooth version outputs a distribution over alignments. This smoothness is crucial to: (i) make the algorithm differentiable and therefore applicable in end-to-end neural network pipelines, and (ii) allow the method to consider multiple hypothesized alignments simultaneously, which we believe to be a beneficial feature early in training.

**Fig. 1.**
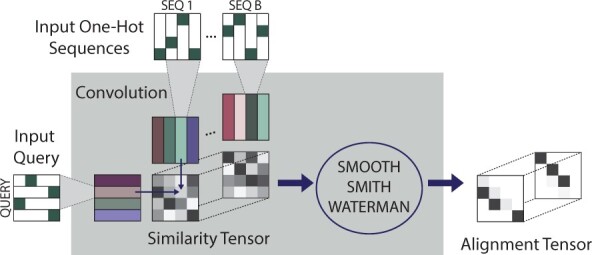
Learned alignment module (LAM). The residues of *B* sequences and a ‘query’ sequence are mapped to vectors using a convolution. For each sequence *k*, an alignment score matrix *a* is computed by taking the dot products of the vectors representing the query sequence and the vectors representing sequence *k*. The similarity tensor is formed by concatenating these matrices, and then our differentiable implementation of smooth Smith–Waterman is applied to each similarity matrix in the tensor to produce an alignment. The resulting *B* smooth pairwise alignments (all aligned to the query sequence) are illustrated as the ‘Alignment Tensor’

We demonstrate the utility of LAM with two differentiable pipelines. First, we design an unsupervised contact prediction method that jointly learns an alignment and the parameters of a Markov Random Field (MRF) for RNA and protein, which we use to infer better structure-based contact maps. Next, we connect our differentiable alignment method to AlphaFold2 [here referred to as AlphaFold, as in Jumper *et al.* (2021)] to jointly infer an alignment that improves its prediction of protein structures. We find that the alignments that improve structure prediction are nonsensical, revealing unexpected behavior of AlphaFold. Our main contributions are as follows:

1. We implemented a smooth and differentiable version of the Smith–Waterman algorithm for local pairwise alignment in JAX (Bradbury *et al.*, 2018). Our implementation includes options for an affine gap penalty, a temperature parameter that controls the relaxation from the highest scoring path (i.e. smoothness), and both global and local alignment settings. Our code is freely available and can be applied in any end-to-end neural network pipeline written in JAX, TensorFlow (Abadi *et al.*, 2015) or via DLPack in PyTorch (Paszke *et al.*, 2019). Moreover, we give a self-contained description of our implementation and its mathematical underpinnings, providing a template for future implementations in other languages.
2. We introduced the LAM, a fully differentiable module for constructing MSAs that is trained in conjunction with a downstream task. For each input sequence, a convolutional architecture produces a matrix of match scores between the sequence and a reference sequence. Unlike a substitution matrix typically input to Smith–Waterman, these scores account for the local *k*-mer context of each residue. Next, we apply our smooth Smith–Waterman (SSW) implementation to these similarity matrices to align each sequence to the reference, yielding an MSA (Fig. 1).
3. We used contact prediction as a case study to demonstrate that joint learning with the LAM can recover alignments that have similar (and sometimes better) performance on contact prediction over traditional methods that input an MSA, establishing that our module works as designed. Our method, *Smooth Markov Unaligned Random Field* (SMURF), takes as input unaligned sequences and jointly learns an MSA (via LAM) and MRF parameters. These parameters can then be used for contact prediction.
4. Finally, we applied the LAM to reveal unexpected behavior of AlphaFold: some low-quality inconsistent alignments yield better structure predictions than sensible alignments of the same sequences. We modify AlphaFold, replacing the input MSA with the output of LAM. For a given set of unaligned, related protein sequences, we backprop through AlphaFold to update the parameters of LAM, maximizing AlphaFold’s predicted confidence. Doing so results in learned MSAs that improve the structure prediction over our initial input MSA for three out of four structures. Despite the improved structure predictions, we find that the MSAs learned by the LAM may be adversarial as indicated by their self-inconsistency. This finding raises questions about how AlphaFold uses the input MSA to make its predictions.

### 1.1 Related work

#### 1.1.1 Differentiable dynamic programming in natural language processing

Differentiable dynamic programming algorithms are needed in order to model combinatorial structures in a way that allows backpropagation of gradients (Berthet *et al.*, 2020; Mensch and Blondel, 2018; Vlastelica *et al.*, 2019). Such algorithms have been used in natural language processing to build neural models for parsing (Durrett and Klein, 2015), grammar induction (Kim *et al.*, 2019), speech (Cai and Xu, 2019) and more. Smooth relaxations of argmax and other non-differentiable functions can enable differentiation through dynamic programs. More generally, Mensch and Blondel (2018) leverage semirings to provide a unified framework for constructing differentiable operators from a general class of dynamic programming algorithms. This work has been incorporated into the Torch-Struct library (Rush, 2020) to enable composition of automatic differentiation and neural network primitives, was recently implemented in Julia (Stock, 2021), and is the basis for our JAX implementation of SSW.

#### 1.1.2 Smooth and differentiable alignment in computational biology

Before end-to-end learning was common, computational biologists used pair HMMs to express probability distributions over pairwise alignments (Durbin *et al.*, 1998; Knudsen and Miyamoto, 2003; Miyazawa, 2000). The forward algorithm applied to a pair HMM can be viewed as a smoothed version of Smith–Waterman. Later, a differentiable kernel-based method for alignment was introduced (Saigo *et al.*, 2006). More recently, Morton *et al.* implemented a differentiable version of the Needleman–Wunsch algorithm for global pairwise alignment (Morton *et al.*, 2020; Needleman and Wunsch, 1970). Our implementation has several advantages: (i) vectorization makes our code faster (Supplementary Fig. S2 and Section S4.3), (ii) we implemented local alignment and an affine gap penalty (Supplementary Section S4.4) and (iii) due to the way gaps are parameterized, the output of Morton *et al.* (2020) cannot be interpreted as an expected alignment (Supplementary Section S4.2). Independent and concurrent work (Llinares-López *et al.*, 2021) uses a different formulation of differentiable Smith–Waterman involving Fenchel-Young loss.

#### 1.1.3 Language models, alignments and MRFs

Previous work combining language model losses with alignment of biological sequences place the alignment layer at the end of the pipeline. Bepler and Berger (2018) first pretrain a bidirectional RNN language model, then freeze this model and train a downstream model using a pseudo-alignment loss. Similarly, Morton *et al.* (2020) use a pretrained language model to parametrize the alignment scoring function. Their loss, however, is purely supervised based on ground-truth structural alignments. Llinares-López *et al.* (2021) use differentiable Smith–Waterman with masked language modeling (MLM) and supervised alignments to learn a scoring function derived from transformer embeddings. For RNA, a transformer embedding has been trained jointly with an MLM and structural alignment (Akiyama and Sakakibara, 2021). In contrast to all of these papers, our alignment layer is in the middle of the pipeline and is trained end-to-end with a task downstream of alignment.

Joint modeling of alignments and Potts models has been explored. Kinjo (2016) include insertions and deletions into a Potts model using techniques from statistical physics. Two other works infer HMM and/or Potts parameters through importance sampling (Wilburn and Eddy, 2020) and message passing (Muntoni *et al.*, 2020), with the goal of designing generative classifiers for protein homology search.

## 2 Materials and methods

### 2.1 Smooth Smith–Waterman

Pairwise sequence alignment is the task of finding an alignment of two sequences with the highest score, where the score is the sum of the ‘match’ scores for each pair of aligned residues and ‘gap’ penalties for residues that are unmatched. The Smith–Waterman algorithm is a dynamic programming algorithm that returns a path with the maximal score. A *smooth* version instead finds a probability distribution over paths in which higher scoring paths are more likely. Smoothness and differentiability can be achieved by replacing the max with logsumexp and argmax with softmax in the dynamic programming algorithm. We implemented an SSW formulation in which the probability that any pair of residues is aligned can be formulated as a derivative (Supplementary Sections S1 and S4). We use JAX due to its JIT (‘just in time’) compilation and automatic differentiation features (Bradbury *et al.*, 2018).

Our speed benchmark indicates that our implementation is faster than the smooth Needleman–Wunsch implementation in Morton *et al.* (2020) for both a forward pass as well as for the combined forward and backward passes (see Supplementary Fig. S2). The latter is relevant when using the method in a neural network pipeline requiring backprogation. Moreover, comparison between a vectorized and naive version of our code shows that vectorization substantially reduces the runtime, see Wozniak (1997) and Supplementary Section S4.3. Vectorization in both sequence length and batch dimension accounts for the speed improvement over the Needleman–Wunsch implementation in Morton *et al.* (2020), which is only vectorized over the batch dimension.

Our SSW has four other features: temperature, affine gap, retrict turns and global alignment. A *temperature* parameter governs the extent to which the distribution concentrated on the highest scoring alignments. In the *affine gap* mode, the first gap in a streak incurs an ‘open’ gap penalty and all subsequent gaps incur an ‘extend’ gap penalty. A *restrict turns* option corrects for the algorithm’s inherent bias toward alignments near the diagonal. We also implemented Needleman–Wunsch to output *global alignments* rather than local alignments. See Supplementary Section S4.4 for additional details of SSW options.

### 2.2 Learned alignment module

The key to improving a Smith–Waterman alignment is finding the right input matrix of alignment scores $a={\left(a_{ij}\right)}_{i\leq \ell_{x},j\leq \ell_{y}}$. Typically, when Smith–Waterman is used for pairwise alignment the alignment score between positions *i* and *j*, *a_ij_*, is given by a BLOSUM or PAM score for the pair of residues *X_i_* and *Y_j_* (Altschul *et al.*, 1997; Dayhoff and Eck, 1972; Henikoff and Henikoff, 1992). This score reflects how likely it is for one amino acid to be substituted for another, but does not acknowledge the context of each residue in the sequence. For example, consider serine, an amino acid that is both small and hydrophilic. In a water-facing part of a protein, serine is more likely to be substituted for other hydrophilic amino acids. In other contexts, serine may only be substituted for other small amino acids due to the geometric constraints of the protein fold. Employing a scoring function with convolutions allows for local context to be considered.

Our proposed LAM adaptively learns a context-dependent alignment score matrix *a_ij_*, performs an alignment based on this score matrix, all *in conjunction with a downstream machine learning task*. The value *a_ij_* expresses the similarity between *X_i_* in the context of $X_{i-w},\ldots X_{i},\ldots X_{i+w}$ and *Y_j_* in the context of $Y_{j-w},\ldots Y_{j},\ldots Y_{j+w}$. We represent position *i* in sequence *X* as a vector $v_{i}^{X}$ obtained by applying a convolutional layer of window size 2w+1 to a one-hot encoding of *X_i_* and its neighbors. The dimension of the vectors is the number of convolutional filters (here 512). The value *a_ij_* in the similarity matrix that we input to Smith–Waterman is the dot product of the corresponding vectors, $a_{ij}=v_{i}^{X}\cdot v_{j}^{Y}.$ To construct an MSA from a reference and *B* other sequences, the LAM constructs a similarity matrix between each sequence and the reference, applies differentiable Smith–Waterman to each similarity matrix, and outputs an alignment of each sequence to the reference (which can be viewed as an MSA) (see Fig. 1). Since this process is entirely differentiable, we can plug the alignment produced by the LAM into a downstream module, compute a loss function, and train the whole pipeline end-to-end.

We confirmed that the similarity scores learned by LAM are much more expressive than BLOSUM scores. Supplementary Figures S6 and S7 illustrate the distribution of similarity scores learned by the LAM when trained in the context of our contact prediction method SMURF. Unlike in the BLOSUM scoring scheme, the score between a pair of amino acids is not simply a function of their identities; instead the score can range substantially depending on the contexts. Moreover, the distribution of scores varies between families.

## 3 Results

### 3.1 Applying the LAM to contact prediction

GREMLIN is a probabilistic model of protein variation that uses the MSA of a protein family to estimate parameters of an MRF (see Supplementary Section S2.1), which in turn are used to predict contact maps (Balakrishnan *et al.*, 2011; Ekeberg *et al.*, 2013; Kamisetty *et al.*, 2013; Ovchinnikov *et al.*, 2014). Since GREMLIN relies on an input MSA, one would expect that improved alignments would yield better contact prediction results. To test this, we designed a pipeline for training a GREMLIN-like model that inputs unaligned sequences and jointly learns the MSA and MRF parameters. We call our method **S**mooth **M**arkov **U**naligned **R**andom **F**ield or SMURF.

SMURF takes as input a family of unaligned sequences and learns both (i) the LAM convolutions and (ii) the parameters of the MRF that are, in turn, used to predict contacts. SMURF has two phases, each beginning with the LAM. First, BasicAlign learns LAM convolutions by minimizing the squared difference between each aligned sequence and the corresponding averaged MSA (Supplementary Fig. S8). This objective (Supplementary Equation S4) encourages alignments where each column is predominantly composed of one or a few specific residues and allows the network to learn convolutions that yield a reasonable alignment before being tasked with deducing pairwise correlations (MRF parameters). These convolutions are then used to initialize the LAM for the second training phase, TrainMRF, where an MLM objective is used to learn MRF parameters and update the convolutions, allowing the network to adjust the alignment (Supplementary Fig. S9). In MLM, random residues in the input are masked and the network uses the energy function described by the MRF parameters to compute a guess (represented as a distribution over residues) for each masked residue. The objective function (Supplementary Equation S6) is a combination of the cross entropy loss of these guesses and regularization terms for the MRF parameters. For further details, see Supplementary Section S2.3.

We compare SMURF to GREMLIN trained with MLM (MLM-GREMLIN) as in Bhattacharya *et al.* (2020). The architecture of MLM-GREMLIN is similar to TrainMRF step of SMURF, except that a fixed alignment is input instead of a learned alignment computed by LAM.

**Fig. 2.**
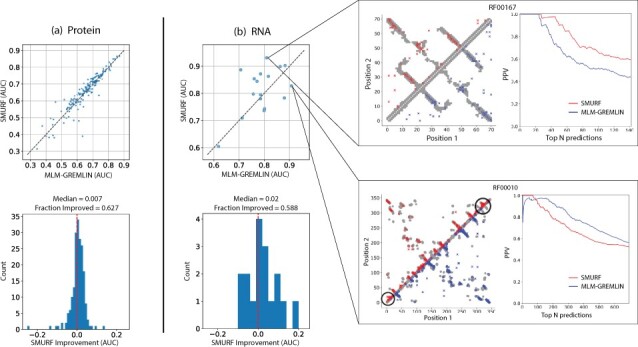
SMURF often outperforms MLM-GREMLIN on (**a**) protein and (**b**) non-coding RNA. (Top) Scatter plots of the AUC of the top L predicted contacts for SMURF versus MLM-GREMLIN. (Bottom) Histograms of the difference in AUC between SMURF and MLM-GREMLIN. (Right) Comparison of contact predictions and the positive predictive value (PPV) for different numbers of top *N* predicted contacts, with *N* ranging from 0 to 2*L*, for SMURF (red, above the diagonal) and MLM-GREMLIN (blue, below the diagonal) for Rfam family RF00010 (Ribonuclease P.) and RF00167 (Purine riboswitch). Gray dots represent PDB-derived contacts, circles represent a true positive prediction, and *x* represents a false positive prediction. For contact predictions for RFAM00010, the black circles highlight a concentration of false positive predictions (A color version of this figure appears in the online version of this article)

We trained and evaluated our model on a diverse set of protein families, as described in Supplementary Section S2.2. Our model was trained separately on each family (i.e. different convolutions are learned for each family), and the families in the training set were used to select the hyper-parameters and network architecture. To evaluate the accuracy of downstream contact prediction, we computed a standard metric used to summarize contact prediction accuracy, i.e. the area under the curve (AUC) for a plot of fraction of top *t* predicted contacts that are correct for *t* equals 1 up to *L*, where *L* is the length of the protein. Figure 2a illustrates that SMURF mildly outperforms MLM-GREMLIN with a median AUC improvement of 0.007 across 193 protein families in the test set. To test whether SMURF requires a deep alignment with many sequences, we ran SMURF on protein families at most 128 sequences. The performance of SMURF and MLM-GREMLIN are comparable even for these families with relatively few sequences, with a median AUC improvement of 0.002 (Supplementary Fig. S11).

Next, we sought to compare qualities of the MSAs learned through SMURF and MSAs fed into GREMLIN, which were generated with HHblits (Steinegger *et al.*, 2019). To quantify the consistency of the MSAs, we compared the BLOSUM scores (Henikoff and Henikoff, 1992) of all pairwise alignments extracted from our learned MSA to those extracted from the HHblits MSA. By this metric, we found that alignments learned by SMURF were more consistent than those from HHblits. Moreover, we observed a slightly positive correlation between increased consistency and contact prediction improvement (Supplementary Fig. S10, left). We also found that SMURF alignments tend to have more positions aligned to the query (Supplementary Fig. S10, right). We hypothesize that this is because our MRF does not have a mechanism to intelligently guess the identity of residues that are insertions with respect to the query sequence (the guess is uniform, see Supplementary Section S2.3).

Next, we applied SMURF to 17 non-coding RNA families from Rfam (Kalvari *et al.*, 2021) that had a corresponding structure in PDB (see Supplementary Section S2.2). Due to the relatively small number of RNAs with known 3D structures, we employed SMURF using the hyperparameters optimized for proteins; fine-tuning SMURF for RNA could improve performance. Overall, we observe that SMURF outperforms MLM-GREMLIN with a median AUC improvement of 0.02 (Fig. 2b). Despite choosing hyperparameters for our network based on protein examples, we see comparatively stronger improvement in RNA. Since our alignments are trained in conjunction with an MRF, covariation patterns inform the alignments. Our observation suggests that there is more to be gained from incorporating covariation into RNA alignment methods as compared to proteins.

In Supplementary Section S5, we further discuss the RNA contact predictions illustrated in Figure 2b and the SMURF predictions for the three most and least improved protein families (Supplementary Figs S12 and S13). We hypothesize that SMURF generates fewer false positive predictions in seemingly random locations because the LAM finds better alignments.

Finally, we performed an ablation study on SMURF (Supplementary Fig. S14). We found that replacing SSW with a differentiable ‘pseudo-alignment’ procedure, similar to Bepler and Berger (2018), degraded performance substantially. Skipping BasicAlign also degraded performance, thus indicating the importance of the initial convolutions found in BasicAlign.

### 3.2 Using backprop through AlphaFold to learn alignments with LAM

Next, we tested whether jointly learning an alignment with AlphaFold could improve structure prediction. While our experiment found this to be possible, the more interesting takeaway was our finding that AlphaFold sometimes makes better predictions from strikingly low-quality alignments as compared to sensible alignments of the same sequences. For our experiment, we selected four CASP14 domains where the structure prediction quality from AlphaFold was especially sensitive to how the MSA was constructed (see Supplementary Section S3.1). We reasoned that the quality was poor due to issues in the MSA and by realigning the sequences using AlphaFold’s confidence metrics we may be able to improve on the prediction quality.

**Fig. 3.**
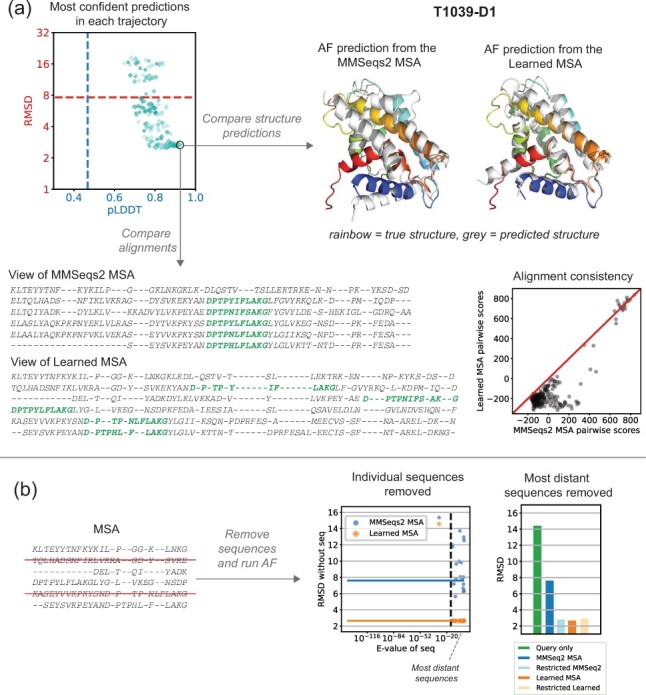
Learned MSA results in improved structure prediction, but a worse alignment for T1039-D1. (**a**) The scatter plot shows the pLDDT and RMSD for the most confident point in each trajectory. The marker color indicates the learning rate ($10^{-2},\;10^{-3},\;10^{-4}$, lightest to darkest) and the shape indicates whether cooling was used (circle = no cooling, square = cooling). The dotted lines show the pLDDT and RMSD of the prediction using the MSA from MMseqs2. We selected the circled point maximizing the confidence (pLDDT) as our ‘Learned MSA’. The native structure is rainbow colored, and the predictions are overlaid in grey. The view of our Learned MSA illustrates the inconsistent alignment of a conserved motif (green) that is aligned accurately in the MMSeqs2 MSA. The scatter plot shows that the pairwise alignment scores for pairs extracted from the Learned MSA are much lower than the scores for pairs extracted from the MMSeqs2 MSA. (**b**) Change in RMSD when individual sequences are removed from the MSA (left) or a group of distant sequences is removed (right) (A color version of this figure appears in the online version of this article)

**Fig. 4.**
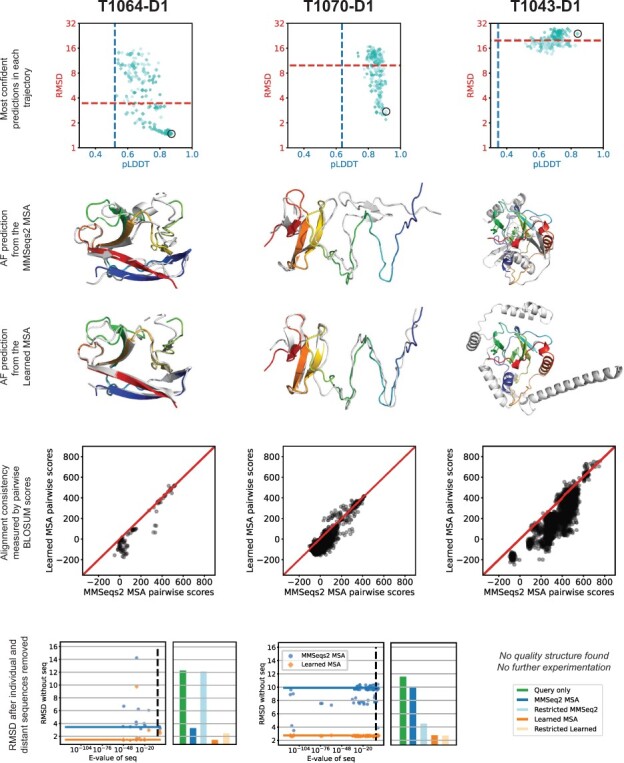
Learned MSA and structure predictions for three additional targets. The plots are analogous to those in Figure 3. An improved structure was found for T1064-D1 and T1070-D1, but not T1043-D1. The MSAs learned for each target were less consistent than their MMSeqs2 counterparts

For each of the four selected CASP targets, separate LAM parameters were fit to maximize AlphaFold’s predicted confidence metrics (see Supplementary Section S3.2). We repeated this 180 times for each target (varying the learning rates, random seeds and smoothness of the alignment), and then selected the learned MSA corresponding to the most confident AlphaFold (AF) prediction as measured by AF’s predicted local Distance Difference Test (pLDDT). For all targets, AF reported higher confidence in the prediction from our learned MSA as compared to the prediction from an MSA with the same sequences generated by MMSeqs2 as implemented in ColabFold (Mirdita *et al.*, 2021). However, only three of the four targets showed an improvement in the structure prediction, as measured by the RMSD (root-mean-squared-distance) to native structure (see Figs 3 and 4).

Next, we compared the learned MSAs that led to better structure predictions to the MMSeqs2 MSAs. Evaluating the learned MSAs by eye, we found our learned MSAs to be strikingly low quality. We saw many examples of inconsistently aligned motifs and even pairs of nearly identical sequences exhibiting completely different alignments with the query. Figure 3a illustrates a conserved motif that is consistently aligned in the MMSeqs2 MSA yet completely scattered in our learned MSA. Next, we designed a method to quantify the quality issues in our learned alignments. We compared the BLOSUM scores (Henikoff and Henikoff, 1992) of all pairwise alignments extracted from our learned MSAs to those extracted from the MMSeqs2 MSA. Indeed, the learned MSAs contain much lower scoring pairwise alignments than those of MMSeqs2 MSAs, indicating far less consistency (Figs 3a and 4), which is the opposite trend we observed for MSAs learned by SMURF. Thus, unlike optimizing the MRF in SMURF, optimizing the confidence of AF predictions does not yield consistent alignments with LAM.

We explored a simple explanation for how low-quality alignments could yield improved structure predictions; perhaps AF uses its axial-like attention to consider only a subset of sequences, and the poor alignments by the other sequences isn’t important or could further disqualify those sequences from being attended to. To investigate this, we evaluated how sensitive the AF predictions are to the inclusion of each individual sequence (Figs 3b and 4). Surprisingly, the prediction accuracy can be incredibly sensitive to the removal of a single sequence, especially for MMSeqs2 MSAs.

Next, we considered the effect of removing subsets of more distant sequences. The MMSeqs2 MSAs were constructed with a lenient *E*-value threshold of 10, which may introduce sequences in the MSA that are not true homologs. For targets T1064-D1 and T1070-D1, we removed all sequences with an *E*-value smaller than $10^{-3}$. The target T1064-D1 has two sequences above this threshold (*E*-values 1.4 and 0.16) that almost certainly are not homologs of the query. (*E*-value, defined as *P*-value multiplied by the size of database, indicates the how many matches with detected similarity are expected to occur by chance alone.) While removing either individually does not substantially change the accuracy of the prediction, removing both worsens the prediction with the MMSeqs2 MSA significantly (RMSD 3.46 to 12.11) and worsens the prediction with our learned MSA mildly (RMSD 1.47 to 2.48). In T1070-D1 we realized the opposite outcome; removing the sequences with *E*-value at least $10^{-3}$ greatly improved the prediction with the MMSeqs2 MSA (RMSD 9.91 to 4.51) and slightly improved the prediction with our learned MSA (RMSD 2.75 to 2.70). Noting the influence of the closest homolog (*E*-value $6.1\times 10^{-30}$) on predictions for T1039-D1, we defined most distant sequences for this target as those with *E*-value greater than $10^{-15}$, leaving only the closest homolog. Restricting to the query and this single homolog improved the MMSeqs2 prediction substantially (RMSD 7.62 to 2.79), bringing it on par with the prediction from our learned MSA on the full set of sequences (RMSD 2.66). The inclusion of this single close homolog is vital; the RMSD of the prediction for the query sequence alone is 11.56.

Finally, we repeated our optimization experiment after removing the distant sequences (Supplementary Fig. S16a). We found that the most confident MSAs learned without the distant sequences tended to yield predictions with similar RMSD to the predictions from the most confident MSAs learned on the full set of sequences (see orange and purple bars in Supplementary Fig. S16b). We also investigated whether it was easier or harder to obtain ‘near optimal’ structure prediction (having an RMSD of 1.25 times the RMSD of the prediction of the learned MSA on the full set) with the restricted set of sequences as compared to the full set. For T1064-D1 our optimization scheme found ‘near optimal’ structures more often with the set of sequences that includes the distant sequences. The opposite was the case for T1039-D1, and there was no strong difference for T1070-D1 (Supplementary Fig. S16b).

## 4 Discussion

In this work, we explored the composition of alignment in a pipeline that can be trained end-to-end without usage of any existing alignment software or ground-truth alignments. With SMURF, we trained alignments jointly with a well-understood MRF contact prediction approach and found mild improvement in accuracy using learned MSAs that were consistent and reasonable. When we instead optimized with AlphaFold’s confidence metrics, we found low-quality MSAs that yielded improved structure predictions for three out of four examples. Our result establishes that in some cases AlphaFold can make accurate structure predictions from very low-quality alignments. Therefore, the task of optimizing AlphaFold structure predictions does not force the LAM to learn high-quality alignments. Perhaps by changing our objective function to also penalize self-inconsistent alignments, we could learn more reasonable MSAs while still improving AlphaFold predictions. Our work both establishes the feasibility of pipelines which jointly learn alignments in conjunction with downstream machine learning systems and highlights the possibility of unexpectedly learning odd alignments when it is not well-understood how exactly the downstream task uses alignments.

While our findings that low-quality, self-inconsistent MSAs can yield improved AlphaFold predictions and that AlphaFold predictions may be quite sensitive to the inclusion of particular sequences may seem paradoxical, these observations reflect behaviors found across deep learning systems. It is well-known that deep neural networks are not robust to adversarial noise (Szegedy *et al.*, 2013). Experiments that use an image recognition neural network to optimize an input image so that the image is confidently classified into a particular category will not necessarily yield a human recognizable image of the category (Mordvintsev *et al.*, 2015; Nguyen *et al.*, 2015). Likewise, when we optimize an input alignment to maximize the confidence of the corresponding AlphaFold prediction, we end up with alignments that are nonsensical (e.g. fail to consistently align a clearly conserved motif, as illustrated in Fig. 3a). Studying adversarial examples has been one approach to trying to understand how neural networks form predictions (Gu and Rigazio, 2014; Heo *et al.*, 2019; Mordvintsev *et al.*, 2015). Our differentiable alignment module could be used with AlphaFold to identify a range of alignments that yield a particular prediction. Studying these alignments could provide insight on which aspects of an alignment are used by AlphaFold to make its prediction.

Our SSW implementation is designed to be usable and efficient, and we hope it will enable experimentation with alignment modules in other applications of machine learning to biological sequences. There is ample opportunity for future work to systematically compare architectures for the scoring function in SSW. The use of convolutions led to relatively simple training dynamics, but other inductive biases induced by recurrent networks, attention mechanisms, or hand-crafted architectures could capture other signal important for alignment scoring. Moreover, training one network across protein families (rather than training a separate network for each family) to produce vector encodings of residues and their contexts could be a promising strategy for aligning arbitrary pairs of protein sequences. We also hope that the use of these more powerful and general scoring functions enables applications in remote homology search, structure prediction, or studies of protein evolution.

Besides MSAs, there are numerous other discrete structures essential to analysis of biological sequences. These include Probabilistic Context Free Grammars used to model RNA Secondary Structure (Nawrocki and Eddy, 2013) and Phylogenetic Trees used to model evolution. Designing differentiable layers that model meaningful combinatorial latent structure in evolution and biophysics is an exciting avenue for further work in machine learning and biology.

## Supplementary Material

btac724_Supplementary_Data

## Data Availability

Links to the data underlying this article are available in our SMURF GitHub repository, at https://github.com/spetti/SMURF.
