# Supplementary material for: End-to-end learning of multiple sequence alignments with differentiable Smith–Waterman
Source: Bioinformatics. 2022 Nov 10;39(1):btac724. doi: 10.1093/bioinformatics/btac724 (PMC9805565; doi:10.1093/bioinformatics/btac724)
Supplement: btac724_Supplementary_Data [file btac724_supplementary_data.pdf]

Supplementary Information

Samantha Petti, Nicholas Bhattacharya, Roshan Rao, Justas Dauparas, Neil Thomas
Juannan Zhou, Alexander M. Rush, Peter Koo, and Sergey Ovchinnikov

1 Smooth and differentiable Smith-Waterman

Pairwise sequence alignment can be formulated as the task of finding the highest scoring path through a directed graph in which edges correspond to an alignment of two particular residues or to a gap. The edge weights are match scores for the corresponding residues or the gap penalty, and the score of the path is the sum of the edge weights. The Smith-Waterman algorithm is a dynamic programming algorithm that returns a path with the maximal score. A *smooth* version instead finds a probability distribution over paths in which higher scoring paths are more likely. We describe a Smooth Smith-Waterman formulation in which the probability that any pair of residues is aligned can be formulated as a derivative.

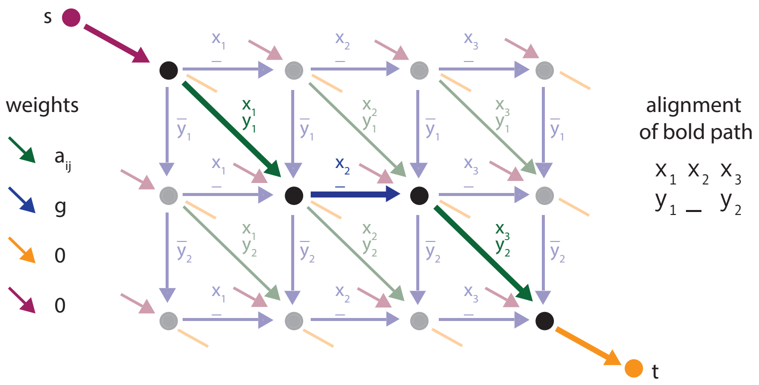

Figure 1: **The alignment graph for sequences $X = x_1x_2x_3$ and $Y = y_1y_2$.** Edge labels describe the corresponding aligned pair, and colors indicate the weights. All red edges start at the source s, and all orange edges end at the sink t. The bold path corresponds to the alignment of X and Y written on the right.

Figure 1 illustrates an alignment graph. For sequences $x_1, x_2, \dots x_{\ell_x}$ and $y_1, y_2, \dots, y_{\ell_y}$, the vertex set contains grid vertices v_{ij} for $0 \le i \le \ell_x$ and $0 \le j \le \ell_y$, a source s, and a sink t. The directed edges are defined so that each path from s to t corresponds to a local alignment of the sequences. The table below describes the definitions, meanings, and weights of the edges.

Edge	Meaning	Weight
$v_{i-1,j-1} \to v_{i,j}$	x_i and y_j are aligned	x_i,y_j alignment score a_{ij}
$v_{i,j-1} \to v_{i,j}$	y_j is aligned with the gap character _	gap penalty g
$v_{i-1,j} \to v_{i,j}$	x_i is aligned with the gap character _	gap penalty g
$s \to v_{i,j}$	x_k for $k \le i$ and y_k for $k \le j$ are excluded	0
$v_{i,j} \to t$	x_k for $k > i$ and y_k for $k > j$ are excluded	0

The Smith-Waterman algorithm iteratively computes the highest score of a path ending at each vertex and returns the highest scoring path ending at t. Let $w(u \to v)$ denote the weight of the edge $u \to v$, and let $N^-(v) = \{u \,|\, u \to v \text{ is an edge}\}$ denote the incoming neighbors of v. Let $f(v)$ be the value of the highest scoring path from s to v. Taking $f(s) = 0$, we compute

$$f(v) = \max_{u \in N^-(v)} \{f(u) + w(u \to v)\}.$$

For grid vertices this simplifies to

$$f(v_{i,j}) = \max\{f(v_{i-1,j-1}) + a_{ij}, f(v_{i,j-1}) + g, f(v_{i-1,j}) + g, 0\}.$$

A path with the highest score is computed by starting at the sink t and tracing backward along the edges that achieve the maxima. (For further explanation see Chapter 2 of Durbin *et al.* (1998) or Smith and Waterman (1981)).

Following the general differentiable dynamic programming framework introduced in Mensch and Blondel (2018), we implement a smoothed version of Smith-Waterman. We compute a smoothed version of the function f, which we denote f^S, by replacing the `max` with `logsumexp`. We again take $f^S(s) = 0$, and define

$$f^S(v) = \log \left(\sum_{u \in N^-(v)} \exp \left(f^S(u) + w(u \to v) \right) \right). \tag{1}$$

We use these smoothed scores and the edge weights to define a probability distribution over paths in G, or equivalently local alignments.

Definition 1. *Given an alignment graph $G = (E, V)$, define a random walk starting at vertex t that traverses edges of G in reverse direction according to transitions probabilities*

$$\mathbb{T}(v \to u) = \frac{\exp\left(f^S(u) + w(u \to v)\right)}{\sum_{u' \in N^-(v)} \exp\left(f^S(u') + w(u' \to v)\right)}$$

and ends at the absorbing vertex s. Let μ_G be the probability distribution over local alignments in which the probability of an alignment A is equal to the probability that the random walk follows the reverse of the path in G corresponding to A.

Under the distribution μ_G, the probability that residues x_i and y_j are aligned can be formulated as a derivative. Mensch and Blondel (2018) describe this relationship in generality for differentiable dynamic programming on directed acyclic graphs. We state their result as it pertains to our context and provide a proof in our notation in Section 4.1.

Proposition 1 (Proposition 3 of Mensch and Blondel (2018))**.** *Let G be an alignment graph and μ_G be the corresponding probability distribution over alignments. Then*

$$\mathbb{P}_{\mu_G}(\ x_i \text{ and } y_j \text{ aligned }) = \frac{\partial f^S(t)}{\partial w(v_{i-1,j-1} \to v_{i,j})} = \frac{\partial f^S(t)}{\partial a_{ij}}.$$

2 SMURF

2.1 GREMLIN details

GREMLIN is a probabilistic model of protein variation that uses the MSA of a protein family to estimate parameters of a MRF of the form

$$\mathbb{P}(X = x) = \frac{1}{Z} \exp\left(E\left(x; v, w\right)\right), \text{where} \quad E\left(x; v, w\right) = \sum_{i=1}^{\ell} \left[v_i(x_i) + \sum_{j=1}^{\ell} w_{ij}(x_i, x_j) \right] \tag{2}$$

and ℓ is the number of columns in the MSA, v_i represents the amino acid propensities for position i, w_{ij} is the pairwise interaction matrix for positions i and j, and Z is the partition function (the value $E(\,\cdot\,; v, w)$ summed over all sequences x). Typically the model is trained by maximizing the pseudo-likelihood of observing all sequences in the alignment (Kamisetty *et al.*, 2013; Ovchinnikov *et al.*, 2014; Ekeberg *et al.*, 2013; Balakrishnan *et al.*, 2011). Here we follow the approach of Bhattacharya *et al.* (2020) and Rao *et al.* (2020) and use *Masked Language Modeling* (MLM) to find the parameters w and v. The pairwise terms w_{ij} can be used to predict contacts by reducing each matrix w_{ij} into a single value that indicates the extent to which positions i and j are coupled.

2.2 Data selection for SMURF

For our analysis of SMURF on proteins, we used the MSAs and contact maps collected in Anishchenko *et al.* (2017). For training and initial tests, we used a reduced redundancy subset of 383 families constructed in Dauparas *et al.* (2019). Each family has least 1K effective sequences, and there is no pair of families with an E-value greater that 1e-10, as computed by an HMM-HMM alignment Hildebrand *et al.* (2009). A random 190 families were used as the training set to identify quality hyperparameters of the model. The remaining 193 families served as the test set and are represented in Main Text Figure 2a, with the exceptions of two outlier families 4X9JA (SMURF AUC = 0.0748, MLM-GREMLIN AUC = 0.0523) and 2YN5A (SMURF AUC = 0.135, MLM-GREMLIN AUC = 0.145). Figure 11 includes data from 99 families from Hildebrand *et al.* (2009) that have at most 128 sequences. A list of the families used in each setting is available in our GitHub repository.

For each non-coding RNA, we aligned the RNA sequence in the PDB along with the corresponding Rfam sequences to an appropriate Rfam covariance model using Infernal (Nawrocki and Eddy, 2013). We then analyzed these sequences using the same procedure outlined for proteins. We evaluated the efficacy of the predicted contact maps using the PDB-derived contact map, where two nucleotides are classified as in contact if the minimum atomic distance is below 8 angstrom. A list of the families used is available in our GitHub repository.

2.3 Details of SMURF

SMURF has two phases: BasicAlign and TrainMRF. Both begin with the learned alignment module (Main Text 1), but they have different architectures and loss functions afterwards.

BasicAlign. Similarity matrices produced by randomly initialized convolutions will produce chaotic alignments that are difficult for the downstream MRF to learn from. The purpose of BasicAlign is to learn initial convolutions whose induced similarity matrices yield alignments with relatively homogeneous columns (see Figure 8). The input to BasicAlign is a random subset of sequences $\mathcal{S} = \{S^{(1)}, \ldots S^{(B)}\}$ in the protein family. A pairwise alignment between each sequence and the first sequence $S^{(1)}$ is produced via the learned alignment module (as described in Main Text Figure 1). This set of alignments can be viewed as an MSA where each column of the MSA corresponds to a position in the first sequence. Averaging the MSA yields

the distribution of residues in each column. Let M_{ix} be the fraction of sequences in $\mathcal{S}$ with residue x aligned to position i of $S^{(1)}$,

$$M_{ix} = \frac{1}{B} \sum_{k=1}^{B} \sum_{j=1}^{\ell_k} p_{ij}^k \mathbb{1}\{S_j^{(k)} = x\}, \tag{3}$$

where ℓ_k is the length of $S^{(k)}$ and p_{ij}^k is the probability that position i of $S^{(1)}$ is aligned to position j of $S^{(k)}$ under the smooth Smith-Waterman alignment. (Note that $\sum_x M_{ix}$ is less than one when there are sequences with a gap aligned to position i of $S^{(1)}$.) The BasicAlign loss is computed by taking the squared difference between each aligned one-hot encoded sequence and the averaged MSA,

$$\mathcal{L}(\mathcal{S}, M) = \sum_{i=1}^{\ell_1} \sum_{x} \sum_{k=1}^{B} \sum_{j=1}^{\ell_k} \left(M_{ix} - p_{ij}^k \mathbb{1}\{S_j^{(k)} = x\} \right)^2 . \tag{4}$$

TrainMRF. In TrainMRF, masked language modeling is used to learn the MRF parameters and further adjust the alignment module convolutions (see Figure 9). The input to TrainMRF is a set of sequences drawn at random from the MSA, $\mathcal{S} = \{S^{(1)}, \ldots S^{(B)}\}$. A random 15% of the residues of the input sequences are masked, and the masked sequences are aligned to the query via the learned alignment module (as described in **??**). The parameters for the alignment module are initialized from BasicAlign, and the query is initialized as the one-hot encoded reference sequence for the family.

The MRF has two sets of parameters: symmetric matrices $w_{ij} \in \mathbb{R}^{A \times A}$ for $1 \le i, j \le \ell_R$ with $w_{ij} = w_{ji}$ that correspond to pairwise interactions of the positions in the reference sequence and position-specific bias vectors $b_i \in \mathbb{R}^A$ for $1 \le i \le \ell_R$. Here ℓ_R denotes the length of the reference sequence, and A is the alphabet size ($A = 20$ for amino acids and $A = 4$ for nucleotides). Unlike traditional parameterizations of a MRF, we do not include gaps in our alphabet. Since our task is reconstructing masked positions in unaligned sequences, we have no need to predict gap characters.

After the sequences are aligned to the query, the infill distribution for each masked position is determined by the MRF parameters as follows. For a masked position j in sequence k, we define $\hat{S}_j^{(k)} \in \mathbb{R}^A$ as the predicted probability distribution over residues at position j of sequence $S^{(k)}$. Let p_{it}^k be the probability that position t of $S^{(k)}$ is aligned to position i of the query under the smooth Smith-Waterman alignment, and let m_t^k be the indicator that position t in sequence $S^{(k)}$ was masked. To compute $\hat{S}_j^{(k)}$, we first compute a score for each residue x that is equal to the expected value (under the smooth alignment) of the terms of the function $E(\,\cdot\,; b, w)$ specific to position j or involving position j and an unmasked position. Then we compute the infill distribution by taking the `softmax`. Formally,

$$\bar{S}_{jx}^{(k)} = \sum_{i=1}^{\ell_R} p_{ij}^k \left(b_{ix} + \sum_{r=1, r \neq i}^{\ell_R} \sum_{t=1}^{\ell_k} p_{rt}^k (1 - m_t^k) w_{ir} \left(x, S_t^{(k)} \right) \right) \quad \text{and} \quad \hat{S}_{jx}^{(k)} = \frac{\exp\left(\bar{S}_{jx}^{(k)}\right)}{\sum_y \exp\left(\bar{S}_{jy}^{(k)}\right)}. \tag{5}$$

The infill distribution is an approximation of how likely each residue is to be present at position j in sequence k if position j were aligned to some position in the query sequence $S^{(1)}$. The approximation considers the values of the linear terms b and the pairwise terms w corresponding only to unmasked positions. (In the case that position j in sequence k is almost certainly an insertion relative to the query sequence $S^{(1)}$, i.e. $\sum_i p_{ij}^k$ is small, our computation will likely provide a poor guess for the residue; in the extreme case where $\sum_i p_{ij}^k = 0$ the infill distribution is uniform over the alphabet. Our model does not have a mechanism to learn the identities of residues that are insertions relative to the query sequence. Ultimately, this is not a concern since we do not use information about insertions to predict the contacts of the query sequence.)

We train the network using a cross entropy loss and $L2$ regularization on w and b with $\lambda = .01$

$$\mathcal{L}(\mathcal{S}, p, b, w) = -\sum_{k=1}^{B} \sum_{j=1}^{\ell_R} \sum_{x} m_j^k S_{jx}^{(k)} \log \hat{S}_{jx}^{(k)} + \frac{\lambda(\ell_R - 1)(A - 1)}{2} \left(\sum_{i,j} \sum_{x,y} w_{ij}(x, y)^2 + \sum_{i=1}^{\ell_r} \sum_{x} b_{ix}^2 \right). \tag{6}$$

After each iteration, the query is updated to reflect the inferred MSA. Let R be the one-hot encoding of the reference sequence. We define C^{i+1} as a rolling weighted average of the MSAs learned through iteration i and Q^i as the query for iteration i,

$$C^1 = R, \qquad C^{i+1} = \eta C^i + (1 - \eta) M^i, \quad \text{and} \quad Q^i = \gamma C^i + (1 - \gamma) R \tag{7}$$

where M^i is the averaged MSA computed as described in Equation (3) from the sequences in iteration i, $\eta = 0.90$, and $\gamma = 0.3$. This process is illustrated by the light blue arrows in Figure 9. Preliminary results on the training set had suggested that updating the query in this manner improved results for some families. However, the ablation study on the test set does not suggest improvement (Figure 14); further investigation is needed to determine the benefits changing the query between iterations.

Once training is complete, we use w to assign a contact prediction score between each pair of positions. The score c_{ij} measures the pairwise interaction between positions i and j, and $\bar{c}_{ij}$ is score after applying APC correction (Dunn *et al.*, 2008),

$$c_{ij} = \left(\sum_{x,y} w_{ij}(x, y)^2 \right)^{1/2} \quad \text{and} \quad \bar{c}_{ij} = c_{ij} - \frac{\sum_k c_{ik} \sum_k c_{kj}}{\sum_{k,\ell} c_{k\ell}}. \tag{8}$$

2.4 SMURF hyperparameter selection

Throughout our hyperparameter search, we kept the following parameters constant: fraction of residues masked at 15%, number of convolution filters at 512, convolution window size at 18, temperature 1.0, regularization λ in Equation (6) at 0.01. Our hyperparameter search consisted of three stages. We initialized the gap penalty as -3 and allowed the network to learn a family-specific gap penalty.

1. First we ran a grid search with on all 190 families in the training set with learning rates $\{.05, 0.10, 0.15\}$, batch sizes $\{64, 128, 256\}$, and iterations {2000 BasicAlign /1000 TrainMRF, 3000 BasicAlign /3000 TrainMRF }. For comparison, we ran MLM-GREMLIN with the same range of learning rates and batch sizes and 3000 iterations. We found that batch size 64 and learning rate 0.05 performed best for MLM-GREMLIN.

2. Then we restricted to a smaller set of families to perform a more extensive hyperparameter search; we included the seven families where MLM-GREMLIN's AUC was less than 0.45 (3AKBA, 3AWUA, 5BY4A, 4C6SA, 3OHEA, 3ERBA, 4F01A) and six families where SMURF consistently performed substantially worse than MLM-GREMLIN (1NNHA, 3AGYA, 4LXQA, 1COJA, 2D4XA, 4ONWA). We considered the following hyperparameter options: learning rates $\{.05, 0.10\}$, batch sizes $\{64, 128, 256\}$, iterations {2000 BasicAlign /1000 TrainMRF, 2000 BasicAlign /2000 TrainMRF, 3000 BasicAlign /1000 TrainMRF }, MSA memory fraction $\eta \in \{0.90, 0.95\}$, and MSA query fraction $\gamma \in \{0.3, 0.5, 0.7\}$.

3. Based on the results of the above hyperparameter search on the select families, we performed a final hyperparameter search on the entire training set. We noticed that performance was better for larger batch sizes, but it was not always possible to run the large batch sizes on our 32 GB GPU for families with longer sequences. For our final hyperparameter search, we used the largest batch size of $\{64, 128, 256\}$ that would fit in memory for each family. We set $\eta = 0.90$, $\gamma = 0.3$, and selected 3000 BasicAlign /1000 TrainMRF iterations because these parameters lead to relatively strong results across the restricted set of families. Learning rate 0.10 outperformed learning rate 0.05 on the restricted set, but learning rate 0.05 generally outperformed learning rate 0.10 in the initial grid search on the full training set. We ran a final test with the aforementioned parameters and the two learning rates on the entire training set, and found that learning rate 0.05 was optimal overall.

   We also ran 4000 iterations of MLM-GREMLIN with predetermined optimal parameters: learning rate 0.05 and batch size 64. We found very similar performance between 3000 and 4000 iterations of MLM-GREMLIN. We chose to compare SMURF to 4000 iterations of MLM-GREMLIN so that both methods were trained for 4000 iterations.

3 AlphaFold experiment

3.1 Data selection for AlphaFold experiment

For our case study, the initial multiple sequence alignments (MSA) were obtained from MMseqs2 webserver as implemented in ColabFold (Mirdita *et al.*, 2021). After trimming the MSAs to their official domain definition, they were further filtered to reduce redundancy to 90 percent and to remove sequences that do not cover at least 75 percent of the domain length, using HHfilter (Steinegger *et al.*, 2019). Continuous domains under 200 in length, with at least 20 sequences, RMSD (root-mean-squared-distance) greater than 3 angstroms and the predicted LDDT (confidence metric) below 75, were selected for the experiment. We include one discontinuous targets T1064-D1 (SARS-CoV-2 ORF8 accessory protein) with only 16 sequences as an extra case study, as this was a particularly difficult CASP target that required manual MSA intervention, guided by pLDDT, to predict well (Jumper *et al.*, 2021a). The filtered MSAs were unaligned (gaps removed, deletions relative to query added back in) and padded to the max length.

3.2 AlphaFold experiment details

AF takes a discrete valued MSA as input and encodes it as a one-hot tensor, which is input into the network. In our work, the input to AF is an amortization of one-hot encoded alignment tensors over the distribution of alignments output by LAM.

We found that the AF predictions were particularly sensitive the the random mask used during evaluation (see Figure 15). For this reason we omitted the mask during evaluation of the MMSeqs2 MSA and throughout our optimization procedure. For simplicity, we considered only one of the five AF models and did not give AF access to the length of insertions relative to the query during our optimization procedure. Our objective function sought to maximize the pLDDT and minimize the alignment error as returned by AF's "model_3_ptm". The AF predictions from the MMSeqs2 MSAs tended to have the overarching structure correct, but were incorrect on certain parts of the sequence. Our goal was for our optimization to correct the incorrect parts of the structure. For this reason we used the more stringent metric of RMSD (rather than the GDT measure of global structure) to evaluate the accuracy of our alignments.

When the number of sequences is low, we find the optimization to be especially sensitive to parameter initialization. To increase robustness, for each target 180 independent optimization trajectories with 100 iterations each were carried out using ADAM. Each trajectory is defined by a random seed, a learning rate $(10^{-2}, 10^{-3}, 10^{-4})$ and whether a cooling scheme was used in Smith-Waterman (temperature 1.0 or temperature decreased linearly from 1.5 to 0.75 across the 100 iterations). In each case, we used 512 convolutional filters, initialized the gap penalty as -3, and allowed the network to learn the gap penalty.

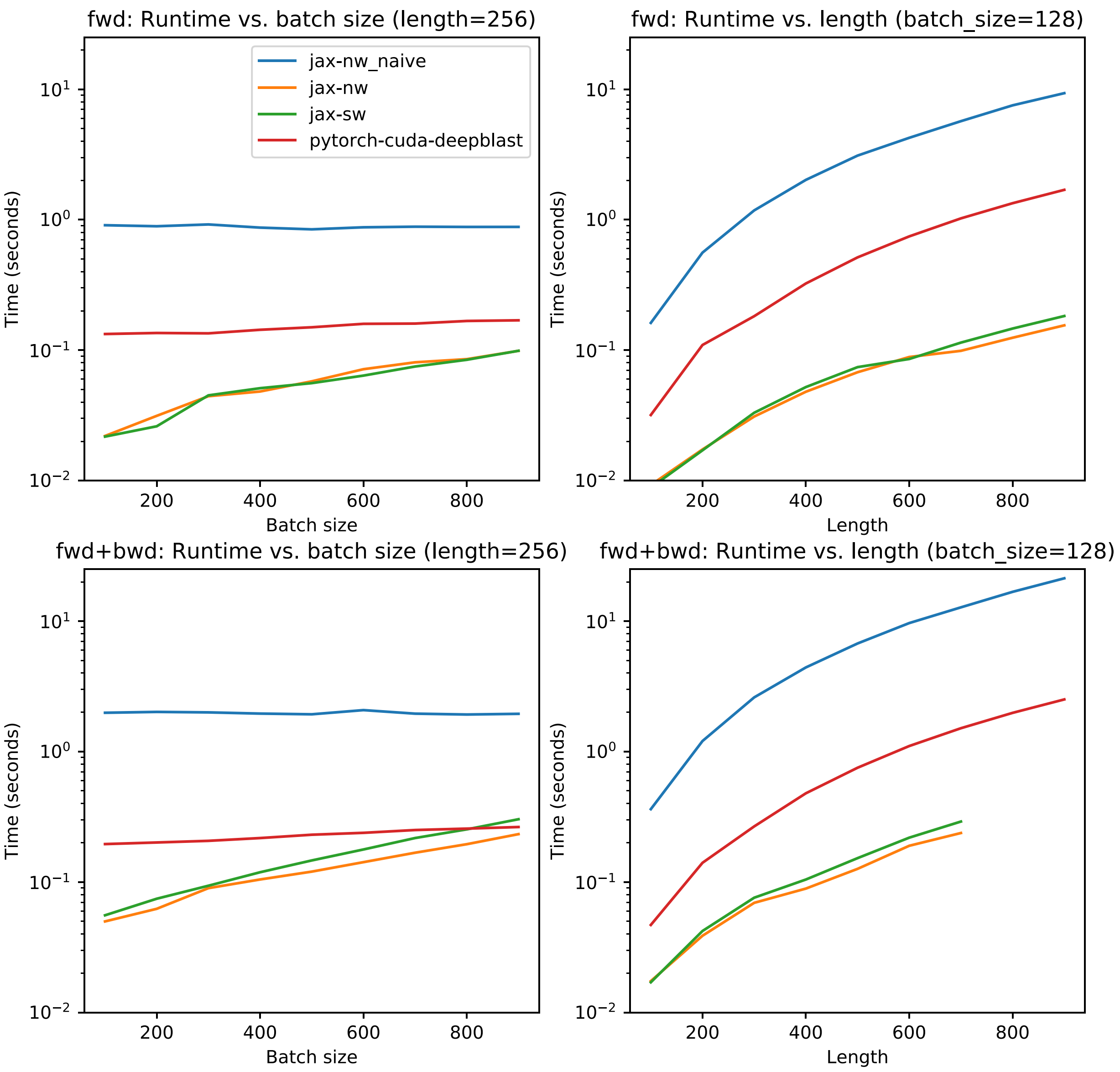

Figure 2: **Runtime comparisons.** We compare the runtimes of the Needleman-Wunsch implementation in Morton *et al.* (2020) our JAX implementations of smooth Smith-Waterman (green), smooth Needleman-Wunsch (orange) and a naive non-vectorized Needleman-Wunsch (blue). Top plots report time for a forward pass, and the bottom plots report time for a forward and backward pass.

4 Smooth Smith-Waterman details and features

4.1 Proof of the probabilistic interpretation of the gradient

For completeness, we now repeat the proof of Proposition 1 given in Mensch and Blondel (2018) for the special case of Smooth Smith-Waterman. Proposition 1 gives a probabilistic interpretation of the gradient $f^S(t)$ with respect to the edge weights a_{ij}. We first give a probabilistic interpretation of the gradient $f^S(t)$ with respect to the vertex scores $f^S(v_{ij})$.

Proposition 2. *Let G be an alignment graph. With respect to the random walk described in Definition 1,*

$$\mathbb{P}(\ v \textit{ is visited }) = \frac{\partial f^S(t)}{\partial f^S(v)}.$$

Proof. Let $N^+(v) = \{u \,|\, v \to u \text{ is an edge in } G\}$ denote the outgoing neighborhood of v. Let $u_1, \dots u_n$ denote the vertices of G in a reverse topological order. We prove the statement by induction with respect to this order. Note $u_1 = t$, and $\mathbb{P}(\ t \text{ is visited }) = \frac{\partial f^S(t)}{\partial f^S(t)} = 1$. Assume that for all $1 \leq i \leq j$, $\mathbb{P}(\ u_i \text{ is visited }) =$

$\frac{\partial f^S(t)}{\partial f^S(u_i)}$. Observe

$$\begin{aligned}
\frac{\partial f^S(t)}{\partial f^S(u_{j+1})} &= \sum_{u' \in N^+(u_{j+1})} \frac{\partial f^S(t)}{\partial f^S(u')} \frac{\partial f^S(u')}{\partial f^S(u_{j+1})} \\
&= \sum_{u' \in N^+(u_{j+1})} \mathbb{P}(\ u' \text{ is visited })\frac{\partial}{\partial f^S(u_{j+1})} \log\left(\sum_{u'' \in N^-(u')} \mathsf{exp}\left(f^S(u'') + w(u'' \to u')\right)\right) \\
&= \sum_{u' \in N^+(u_{j+1})} \mathbb{P}(\ u' \text{ is visited })\frac{\mathsf{exp}\left(f^S(u_{j+1}) + w(u_{j+1} \to u')\right)}{\sum_{u'' \in N^-(u')} \mathsf{exp}\left(f^S(u'') + w(u'' \to u')\right)} \\
&= \sum_{u' \in N^+(u_{j+1})} \mathbb{P}(\ u' \text{ is visited })\mathbb{T}\left(u' \to u_{j+1}\right) \\
&= \mathbb{P}(\ u_{j+1} \text{ is visited }),
\end{aligned}$$

where in the second equality we apply the inductive hypothesis. □

Proof of Proposition 1. It suffices to show that for each directed edge $u \to v$ in G

$$\frac{\partial f^S(t)}{\partial w(u \to v)} = \mathbb{P}(\ \text{edge } u \to v \text{ is traversed })$$

where the traversal occurs from v to u in the random walk. Observe

$$\begin{aligned}
\frac{\partial f^S(t)}{\partial w(u \to v)} &= \frac{\partial f^S(t)}{\partial f^S(v)} \frac{\partial f^S(v)}{\partial w(u \to v)} \\
&= \mathbb{P}(\ v \text{ is visited })\frac{\partial}{\partial w(u \to v)} \log\left(\sum_{u' \in N^-(v)} \mathsf{exp}\left(f^S(u') + w(u' \to v)\right)\right) \\
&= \mathbb{P}(\ v \text{ is visited })\frac{\mathsf{exp}\left(f^S(u) + w(u \to v)\right)}{\sum_{u' \in N^-(v)} \mathsf{exp}\left(f^S(u') + w(u' \to v)\right)} \\
&= \mathbb{P}(\ v \text{ is visited })\mathbb{T}\left(v \to u\right) \\
&= \mathbb{P}(\ \text{edge } u \to v \text{ is traversed }).
\end{aligned}$$

□

4.2 Difference in Needleman-Wunsch implementation of Morton et. al.

Morton *et al.* (2020) implement a differentiable version of the Needleman-Wunsch global alignment algorithm (Needleman and Wunsch, 1970). Their implementation differs from ours in how gaps are parameterized. Consequently, their output indicates where gaps or matches are likely, whereas our output expresses matches in an expected alignment.

Morton *et al.* (2020) define

$$v_{i,j} = \mu_{i,j} + \max_{\Omega}\left(v_{i-1,j-1}, g_{i,j} + v_{i-1,j}, g_{i,j} + v_{i,j-1}\right),$$

where $g_{i,j}$ is the gap penalty for an insertion or deletion at i or j, $\mu_{i,j}$ is the alignment score for X_i and Y_j, and $\max_\Omega(x) = \log\left(\sum_i \mathsf{exp}\left(x_i\right)\right)$ (see Appendix A of Morton *et al.* (2020)). The values $v_{i,j}$ are analogous to our definition f^S on grid vertices (Equation (1)) with match scores $\mu_{i,j} = a_{i,j}$,

$$f^S(v_{i,j}) = \max_{\Omega}\left(f^S(v_{i-1,j-1}) + \mu_{i,j}, f^S(v_{i,j-1}) + g, f^S(v_{i-1,j}) + g\right).$$

In the alignment graph for their formulation, gap edges have weight $\mu_{i,j} + g_{i,j}$. In our alignment graph, gap edges have weight g; the match score $\mu_{i,j}$ does not play a role, and our gap penalty is not position dependent.

Their code outputs the derivatives $\frac{\partial v_{N,M}}{\partial \mu_{i,j}}$. The derivative $\frac{\partial v_{N,M}}{\partial \mu_{i,j}}$ is high whenever the dominant alignment path uses an edge whose weight includes $\mu_{i,j}$; this includes the edges that corresponds to gaps. In contrast, in our formulation $a_{i,j} = \mu_{i,j}$ appears on the match edge only, and so $\frac{\partial f^S(t)}{\partial a_{i,j}}$ is high only when the dominant alignment path uses the edge corresponding to a match. Proposition 1 establishes that $\frac{\partial f^S(t)}{\partial a_{i,j}}$ equal to the probability that X_i and Y_j are aligned, so our output is an expected alignment. Figure 3 establishes that this is not the case for the output of the Needleman-Wunsch implementation of Morton *et al.* (2020).

4.3 Vectorization in our SSW implementation

Following the approach of Wozniak (1997), we implement a version of smooth Smith-Waterman where the values on the anti-diagonal are computed simultaneously. The vectorization speeds up our code substantially. In order to compute the final score $f^S(t)$, we iteratively compute the scores of the grid vertices $f^S(v_{i,j})$, which take as input the values $f^S(v_{i-1,j}), f^S(v_{i,j-1})$, and $f^S(v_{i-1,j-1})$. In a simple implementation, a `for` loop over i and j is used to compute the values $f^S(v_{i,j})$ (Figure 4a). To leverage vectorization, we instead compute the values $f^S(v_{i,j})$ along each diagonal in tandem, i.e. all (i,j) such that $i + j = d$. To implement this, we rotate the matrix that stores the values $f^S(v_{i,j})$ by 45 degrees so that each diagonal now corresponds to a row (see Figure 4b). In the rotated matrix, the values in a row d are a function of the values in rows $d-1$ and $d-2$, and therefore we can apply vectorization to quickly fill the matrix.

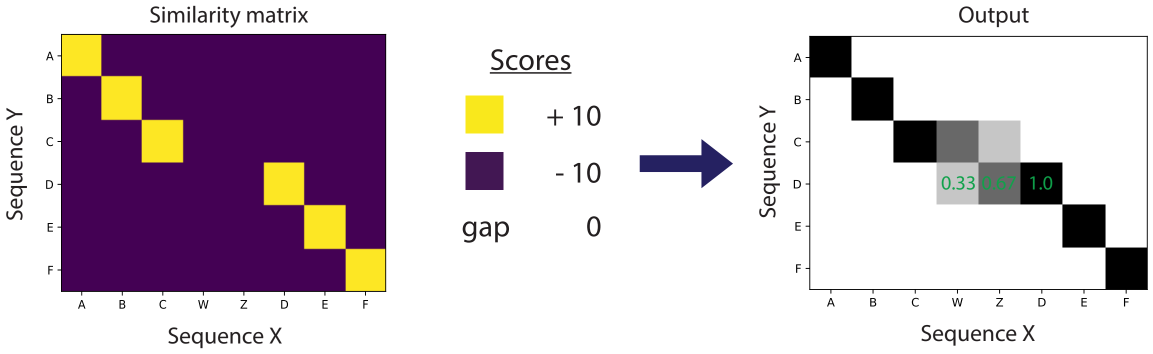

Figure 3: **The output of the Needleman-Wunsch implementation of Morton *et al.* (2020) is not an expected alignment.** It is not the case that $Y_4 = D$ is aligned with $X_4 = W$ with probability 0.33, $X_5 = Z$ with probability 0.67, and $X_6 = D$ with probability 1.0 because in any alignment, Y_4 can be aligned to at most one residue of sequence X.

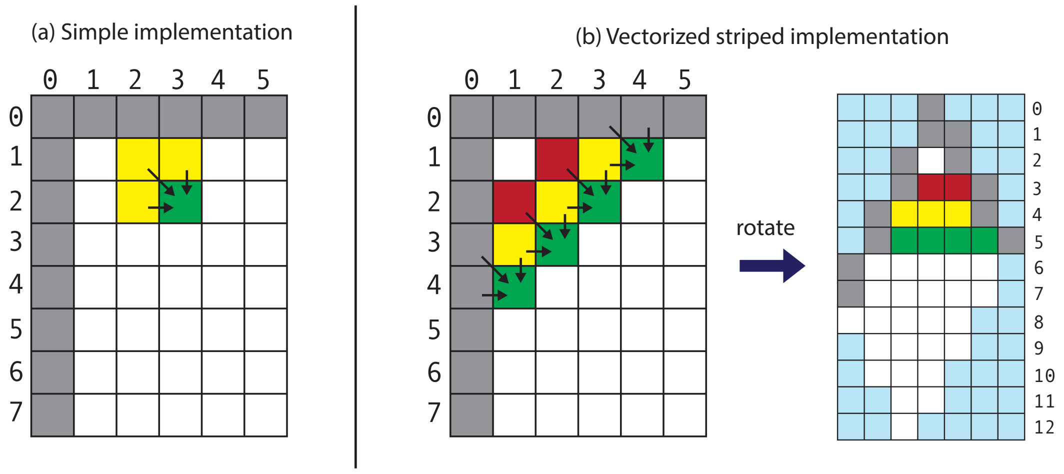

Figure 4: **Vectorized implementation.** (a) In a simple implementation, the value $f^S(v_{i,j})$ are computed individually in a `for` loop over i and j. (b) In an anti-diagonal implementation, the values along each diagonal in the matrix are computed in tandem. We implement this with vectorization by rotating the matrix and computing the values in each row in tandem. The blue denotes meaningless positions in the rotated matrix that we set to $-\infty$. This figure is inspired by Michael Brudno (University of Toronto).

4.4 SSW options

Our smooth Smith-Waterman implementation has the following four additional options.

Temperature parameter. The temperature parameter T controls the extent to which the probability distribution over alignments is concentrated on the most likely alignments; higher temperatures yield less concentrated alignments. We compute the smoothed score for the vertex v as

$$f^S(v) = T \cdot \log\left(\sum_{u \in N^-(v)} \exp\left(\frac{f^S(u) + w(u \to v)}{T}\right)\right),$$

which matches Equation (1) at the default $T = 1$.

Affine gap penalty. The "affine gap" scoring scheme introduced to Smith-Waterman by Gotoh (1982) applies an "open" gap penalty to the first gap in a stretch of consecutive gaps and an "extend" gap penalty to each subsequent gap. The open gap penalty is usually larger than the extend penalty, thus penalizing length L gaps less severely than L separate single residue gaps.

To implement an affine gap penalty, we use a modified alignment graph with three sets of grid vertices that keep track of whether the previous pair in the alignment was a gap or a match. Edges corresponding to the first gap in a stretch are weighted with the "open" gap penalty[1]. Figure 5a illustrates the incoming edges of the three grid vertices for (i, j). Paths corresponding to alignments with x_i and y_j matched pass through v^D_{ij}, paths corresponding to alignments with a gap at x_i pass through v^L_{ij}, and paths corresponding to alignments with a gap at y_j pass through v^T_{ij}. Storing three sets of grid vertices requires three times the memory used by the version with a linear gap penalty. For this reason we implemented SMURF with a linear gap penalty.

Restrict turns. Smooth Smith-Waterman is inherently biased towards alignments with an unmatched stretch of X followed directly by an unmatched stretch of Y over alignments with an equally long unmatched stretch in one sequence. Consider the example illustrated in Figure 5b where the highest scoring match states are depicted by bold black, light blue, and dark green lines. Suppose the match scores of the light blue and

[1]By convention, we charge the open gap penalty when a gap in sequence X is proceeded by a gap in sequence Y and vice versa.

the dark green are identical. With a standard Smith-Waterman scoring scheme (no affine gap), the alignment containing the black and light blue segments has the same score as each alignment containing the black and dark green segments. However, there are more alignments that pass through the dark green segment. There are ten ways to align ABC and VW with no matches (the red, purple, orange, brown, and light green paths illustrate five such ways), but only one way to align $VWXYZ$ with gaps (navy blue). Smooth Smith-Waterman will assign the same probability to each of these paths. However, since ten of the eleven paths go through the dark green segment, the expected alignment output by smooth Smith-Waterman will favor the dark green segment. This bias becomes more pronounced the longer the segments; there are $\binom{L}{A}$ alignments of a sequence of length L and a sequence of length $L - A$ with no matches.

To remove this bias, we implemented "restrict turns" option that forbids unmatched stretches in the X sequence from following an unmatched stretch in the Y sequence. To do so, we again use an alignment graph with three sets of grid vertices to keep track of the previous pair in the alignment. Removing the edge with the asterisk in Figure 5a, forbids transitions from an unmatched stretch in the Y sequence to an unmatched stretch in the X sequence. When implemented with this restrict turns option, smooth Smith-Waterman will find exactly one path through the dark green and black segments in Figure 5: the path highlighted in red. Due to the increased memory requirement of the restrict turn option, we did not utilize the option in SMURF.

Global Alignment. We also implement the Needleman-Wunsch algorithm, which outputs global alignments rather than local alignments.

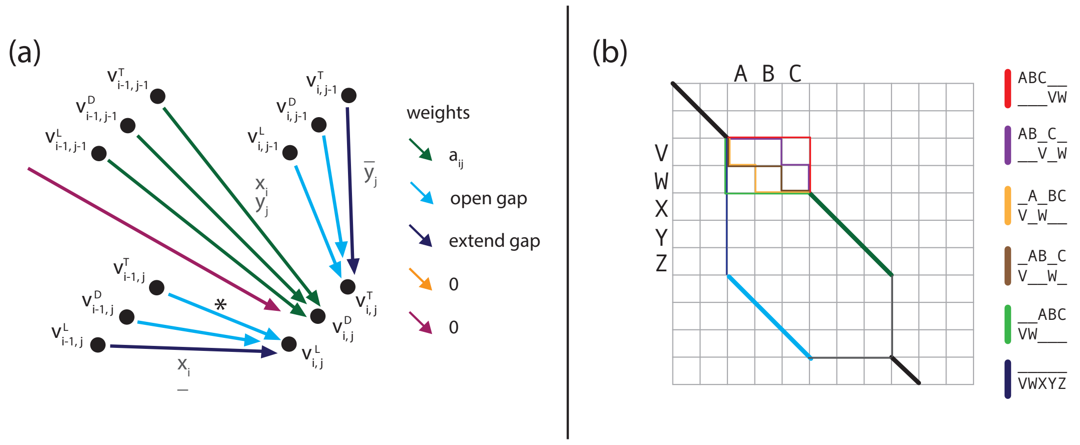

Figure 5: **Algorithm modification for the affine gap penalty and restrict turns options.** (a) The modification of the alignment graph from Figure 1 needed for the affine gap penalty. Incoming edges of the vertices v_{ij}^L,v_{ij}^D, and v_{ij}^T are illustrated. The colors of the edges indicate their weights. The grey labels describe the corresponding aligned pair for each group of edges. The red edge is incoming from the source vertex s. There is an outgoing edge from v_{ij}^D to the sink t for all $i, j \geq 1$ (not pictured). The edge marked with an asterisk is removed under the "restrict turns" option. (b) Without the restrict turns option, there ten paths containing both black segments and dark green segment. The red, purple, orange, brown, and light green illustrate five of these paths. There is only one path that contains both black segments and light blue segment, as depicted in navy blue. The sub-alignments corresponding to the colored segments are written on the right. With the restrict turns option the purple, orange, brown, and green paths are not valid.

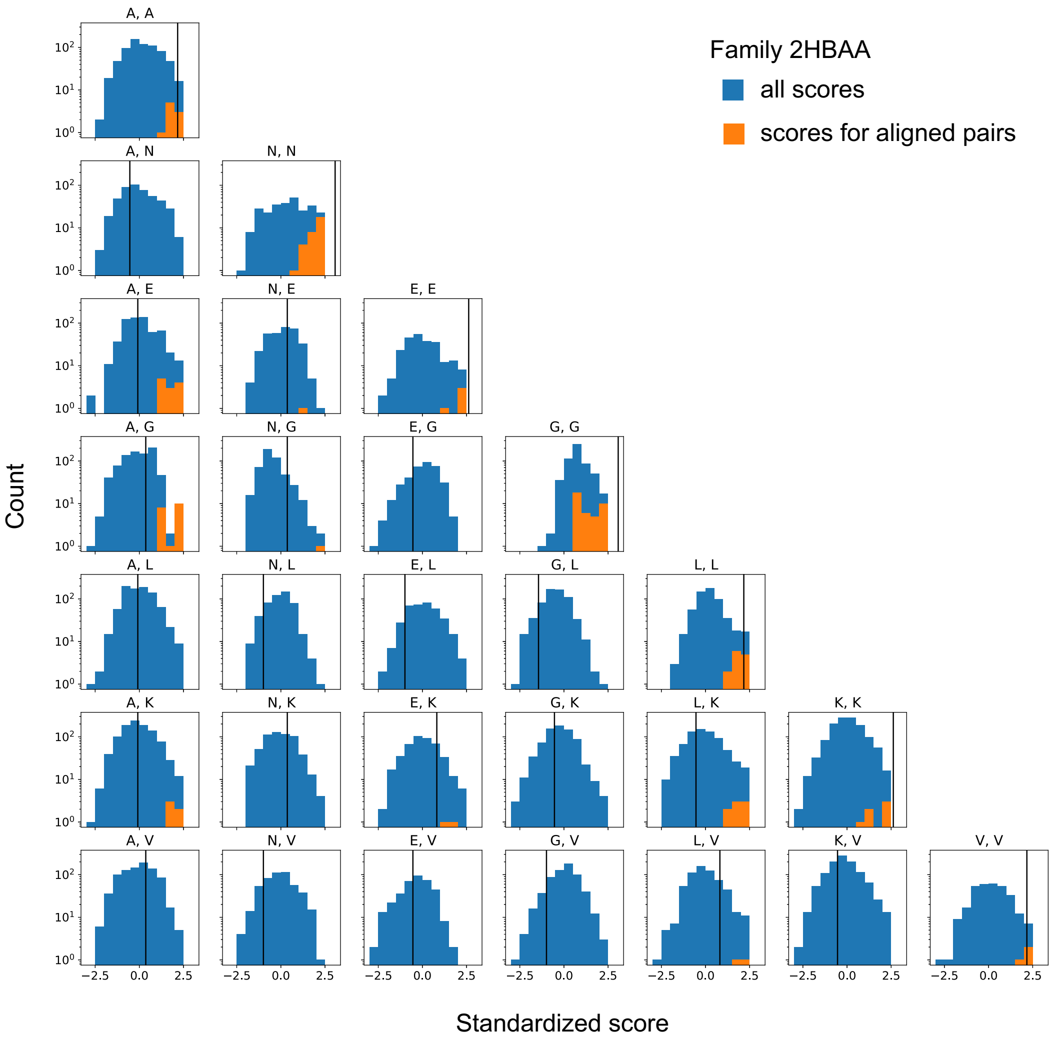

Figure 6: **Distribution of learned similarity scores by amino acid pairs for 2HBAA.** Blue: histograms of standardized similarity scores input into SSW in the LAM for protein family 2HBAA by identity of amino acid pair. Orange: overlaid historgram of scores restricted to pairs of residues with expected value of alignment at least 0.5. All scores are standardized by subtracting the mean and dividing by the standard deviation, computed over all similarity scores between 24 sequences and the query (i.e. values in the similarity tensor illustrated in Main Text Figure 1). The black lines represent the BLOSUM scores for the pair of amino acids, standardized in the same manner. Only amino acids that are present at least four times in the query or on average at least four times among the 24 input sequences are included in the figure.

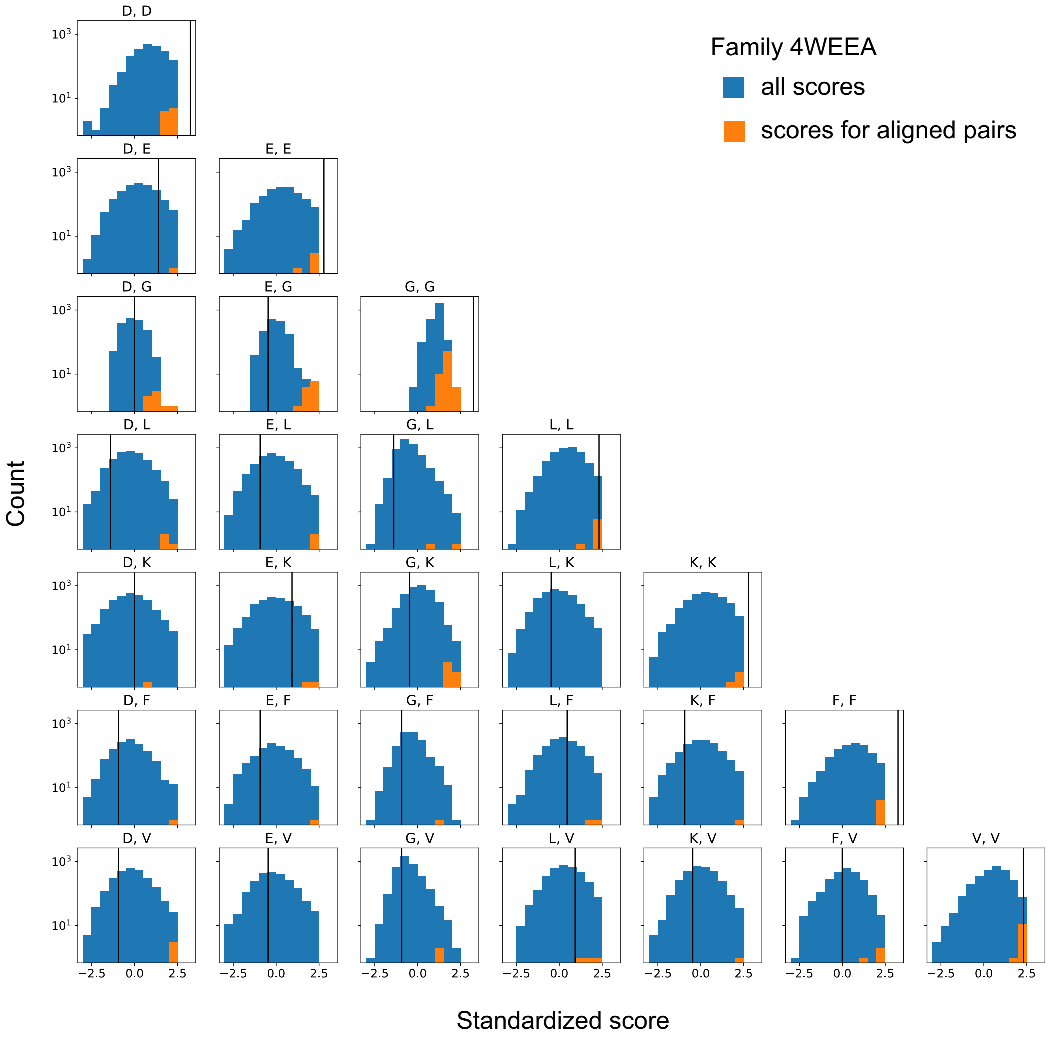

Figure 7: **Distribution of learned similarity scores by amino acid pairs for 4WEEA.** Analogous plot to Figure 6. Only amino acids that are present at least nine times in the query or on average at least nine times among the 24 input sequences are included in the figure.

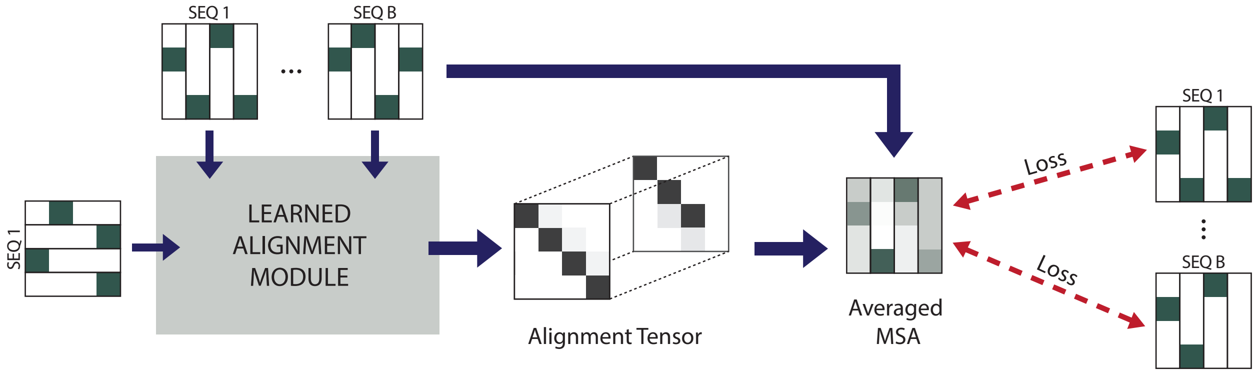

Figure 8: **BasicAlign.** An alignment is computed with the learned alignment module (Main Text Figure 1), and the corresponding MSA is averaged. Squared loss (Equation (4)) is computed between the averaged MSA and the one-hot encoding of the aligned input sequences.

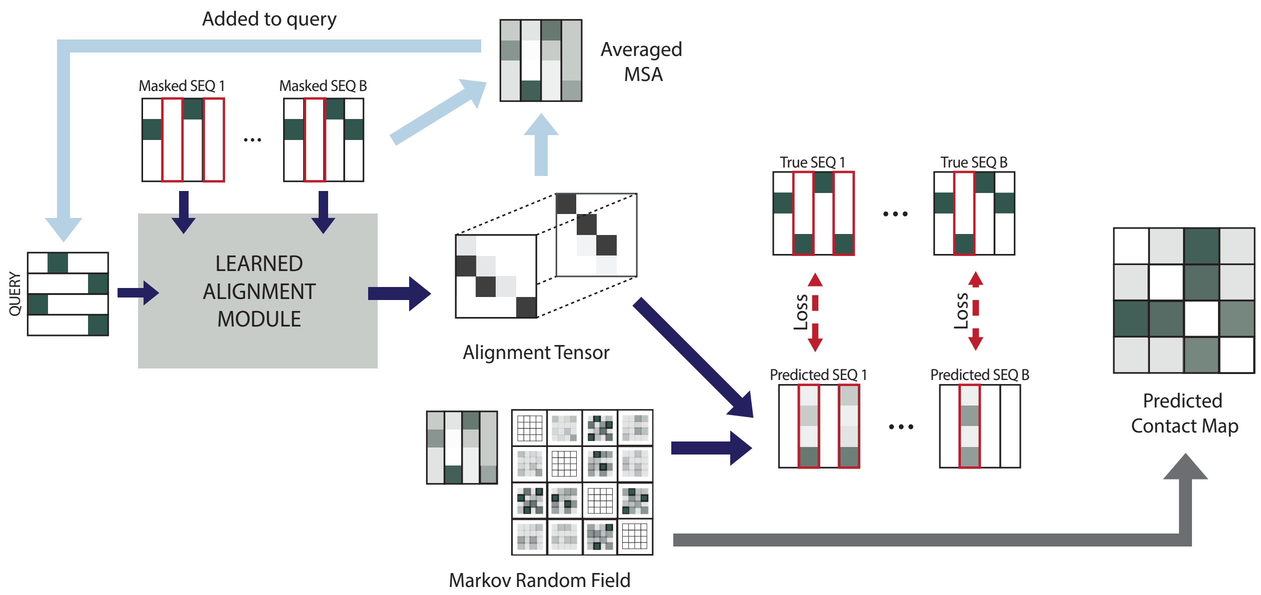

Figure 9: **TrainMRF.** Random positions in the input sequences are masked, then aligned with the LAM (Main Text Figure 1). A prediction for the masked positions is computed from the MRF parameters according to Equation (5). The network is trained with cross entropy loss given by Equation (6). The light blue arrows illustrate the update to the query that occurs between iterations of training; the query is a weighted average of the one-hot query sequence and a running average of the MSAs computed in previous iterations, see Equation (7). The grey arrow depicts the extraction of the contact map from the MRF matrix w at the end of training, as described in Equation (8).

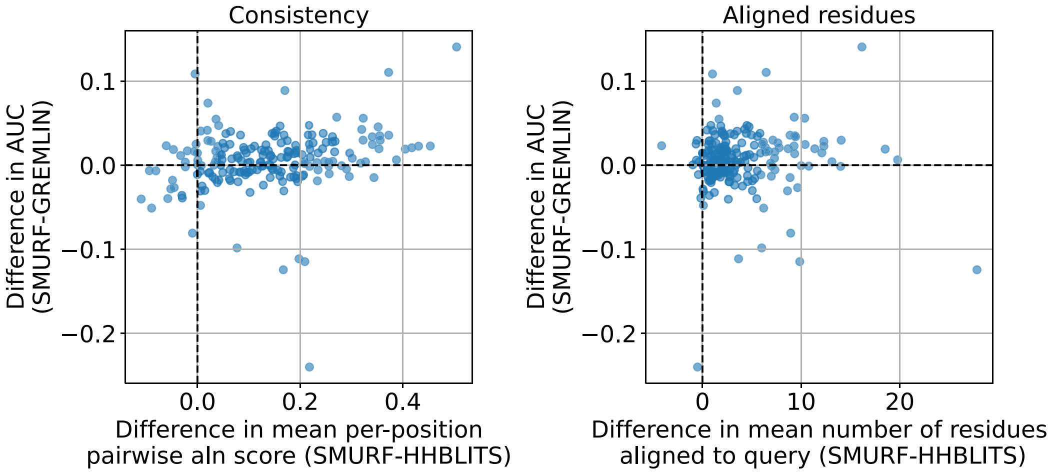

Figure 10: **SMURF-learned alignments are more consistent and have more residues aligned to the query in comparison to HHBlits alignments.** Left: The BLOSUM pairwise alignment scores are on average higher for SMURF MSAs as compared to HHBlits MSAs. There is a postive correlation between an increase in pairwise alignment score and the improvement of SMURF over GREMLIN contact accuracy prediction. BLOSUM scores were computed only over positions that correspond to a residue in query sequence and used an affine gap penalty with open penalty -11 and extend penalty -1. Right: SMURF MSAs tend to have more positions aligned to the query as compared to HHBlits MSAs. This quantity does not appear correlated with the relative performance of SMURF over GREMLIN. Both plots were generated from a random sample of 50 sequences from each alignment (out of 1024 sequences).

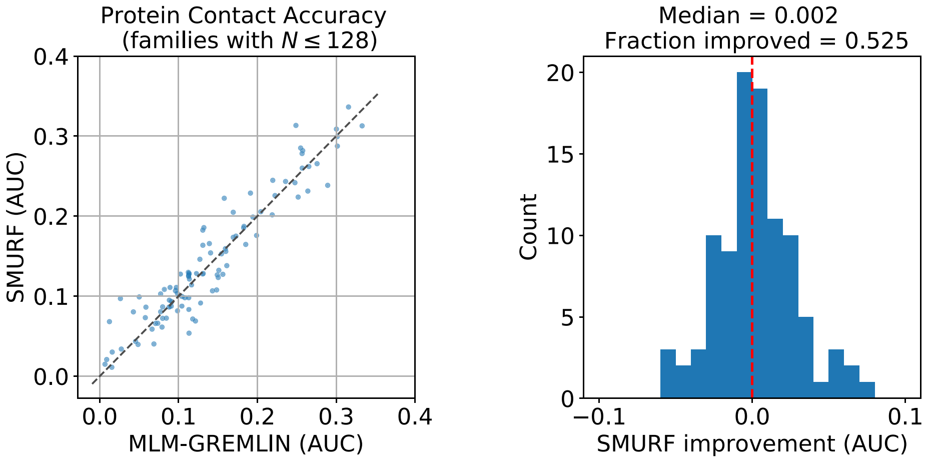

Figure 11: **SMURF performance on 99 protein families from Anishchenko *et al.* (2017) with at most 128 sequences.** Left: Scatter plot of the AUC of the top L predicted contacts for SMURF versus MLM-GREMLIN. Right: Histogram of the difference in AUC between SMURF and MLM-GREMLIN.

5 Further analysis of example SMURF predictions

5.1 RNA contact prediction.

By comparing the positive predictive value (PPV) for different numbers of predicted contacts, we see that SMURF consistently yields a higher PPV for RFAM family RF00167 (Main Text 2b). For RF00010, it starts off higher but then drops off faster, leading to a lower overall AUC. Upon a visual inspection of the contact predictions, MLM-GREMLIN evidently generates more false positive predictions in seemingly random locations. On the other hand, SMURF largely resolves this issue, even for RF00010, presumably as a result of a better alignment. Interestingly, SMURF's lower AUC for RF00010 can be attributed to a concentration of false positive predictions near the 5' and 3' ends. It remains unclear whether these represent a coevolution-based structural element that was not present in the specific RNA sequence deposited in PDB or whether these arise from artifacts of the learned alignment.

5.2 Protein contact prediction and alignments.

Next, we investigated the contact predictions and alignments produced by SMURF. Figure 12 and Figure 13 illustrate the contact predictions, corresponding positive predictive value (PPV) plots, and alignments for the three families that improved the most and least (respectively) under SMURF as compared to MLM-GREMLIN. The poor performance of SMURF on 3LF9A can be attributed to the misalignment of the first ≈ 25 residues of many sequences (including the one illustrated) to positions ≈ 75 to 100 of the reference rather than to the first 25 positions of the reference. This is likely because the gap penalty for leaving positions ≈ 25 to 75 unaligned outweighs the benefit of aligning to beginning of the reference. Since our code computes a local alignment, there is no penalty for leaving positions at the beginning of the reference unaligned. Perhaps using our implementation of Smith-Waterman with an affine gap penalty would lead the network to learn a less severe penalty for long gaps and arrive at correct alignment. For the most improved families, we see that SMURF tends to predict fewer false positive predictions in seemingly random positions, as observed for RNA.

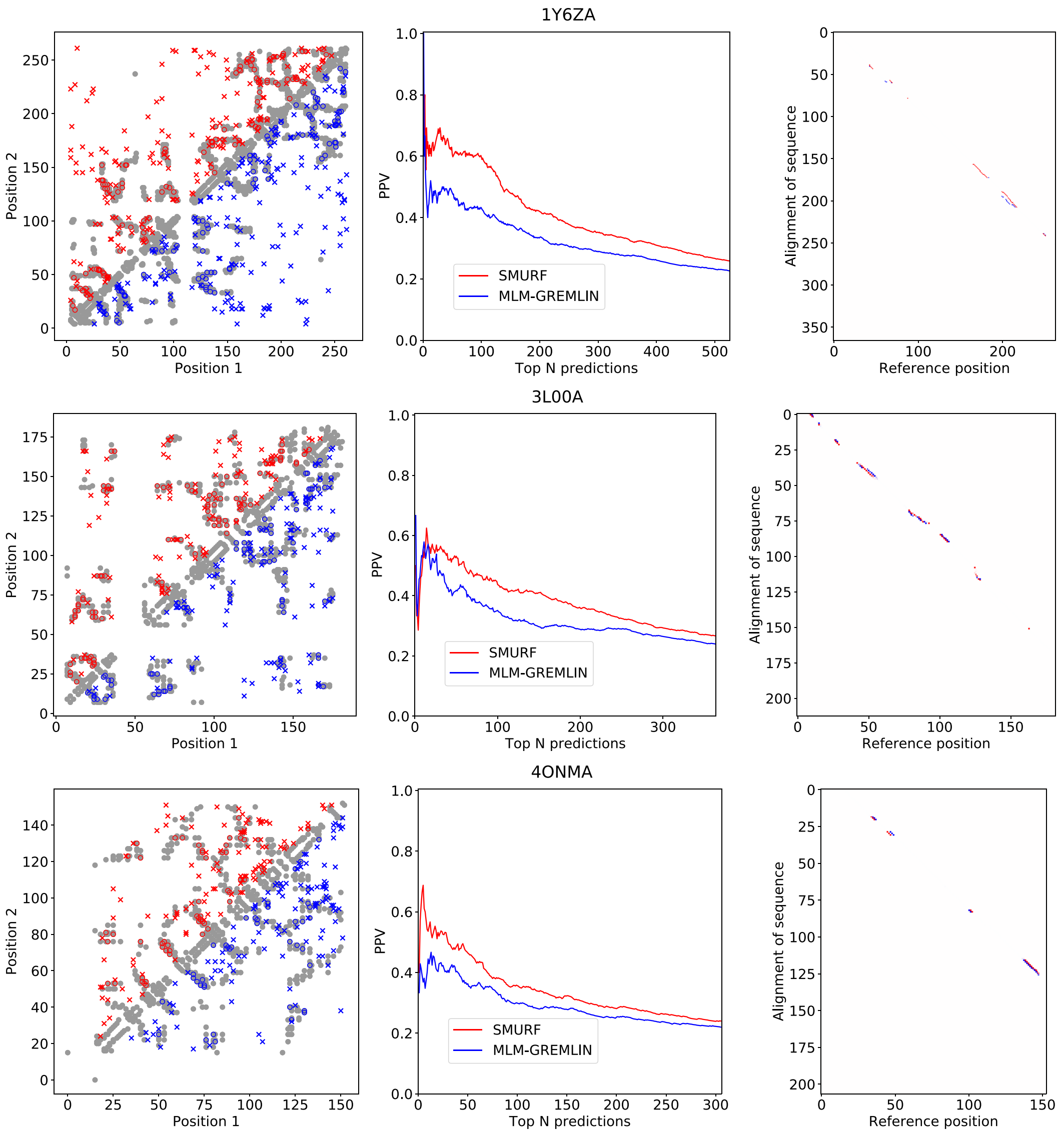

Figure 12: **Contact predictions and alignments for the three most improved protein families.** Left: Comparison of contact predictions between SMURF (red) and MLM-GREMLIN (blue). Gray dots represent PDB-derived contacts, circles represent a true positive prediction, and x represents a false positive prediction. Middle: The positive predictive value (PPV) for different numbers of top N predicted contacts, with N ranging from 0 to $2L$. Right: Comparison of the alignment of a random sequence in the family to the reference sequence. Red indicates aligned pairs that appear in the SMURF alignment, but do not appear in the given alignment. Blue indicate aligned pairs that appear in the given alignment, but do not appear the alignment found by SMURF.

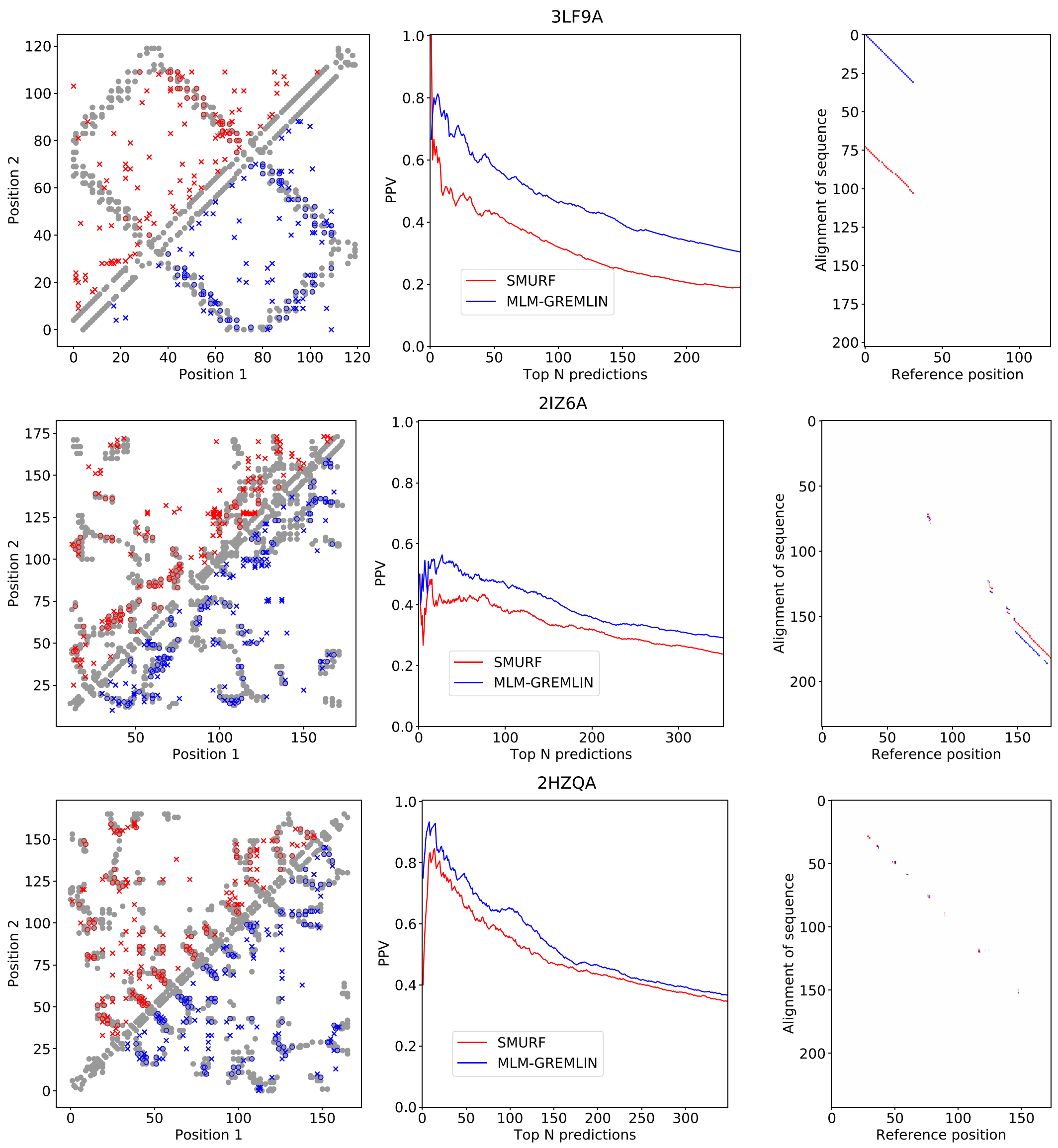

Figure 13: **Contact predictions and alignments for the three worst performing protein families (as compared to MLM-GREMLIN).** Left: Comparison of contact predictions between SMURF (red) and MLM-GREMLIN (blue). Gray dots represent PDB-derived contacts, circles represent a true positive prediction, and x represents a false positive prediction. Middle: The positive predictive value (PPV) for different numbers of top N predicted contacts, with N ranging from 0 to $2L$. Right: Comparison of the alignment of a random sequence in the family to the reference sequence. Red indicates aligned pairs that appear in the SMURF alignment, but do not appear in the given alignment. Blue indicate aligned pairs that appear in the given alignment, but do not appear the alignment found by SMURF.

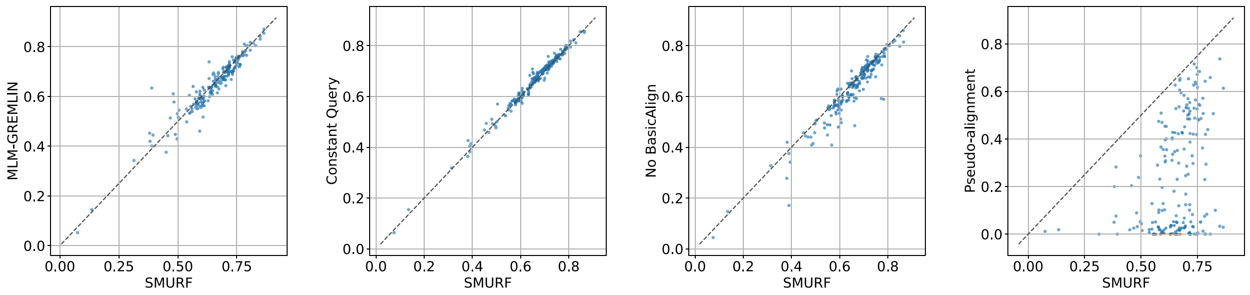

Figure 14: **Ablation results.** Contact AUC for SMURF versus ablated methods. Each point represents one family in the test set. In "Constant Query," we did not update the the query with the averaged MSA between iterations (as depicted by light blue arrows in Figure 9). In "No BasicAlign," the convolutions were not initialized with BasicAlign, and instead TrainMRF was run for 4000 iterations. In "pseudo-alignment," we replaced Smith-Waterman with a pseudo-alignment obtained by taking the softmax of the similarity matrix row-wise and column-wise, multiplying the resultant matrices, and taking the square root (similar to Bepler and Berger (2018)).

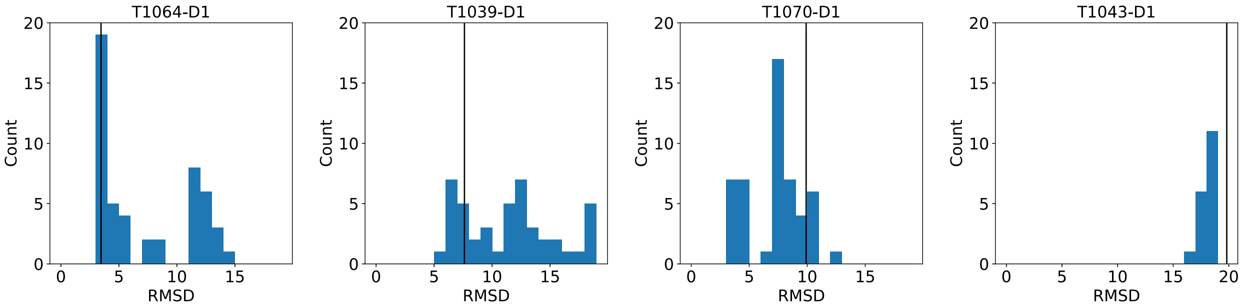

Figure 15: **Sensitivity of AlphaFold predictions to random masking.** By default, a random mask is used when AlphaFold makes a structure prediction (Jumper *et al.*, 2021b). The distribution of RMSD of AlphaFold predictions for MMSeqs2 MSAs with different random seeds used for the masks. The black line shows the RMSD of the prediction without the mask.

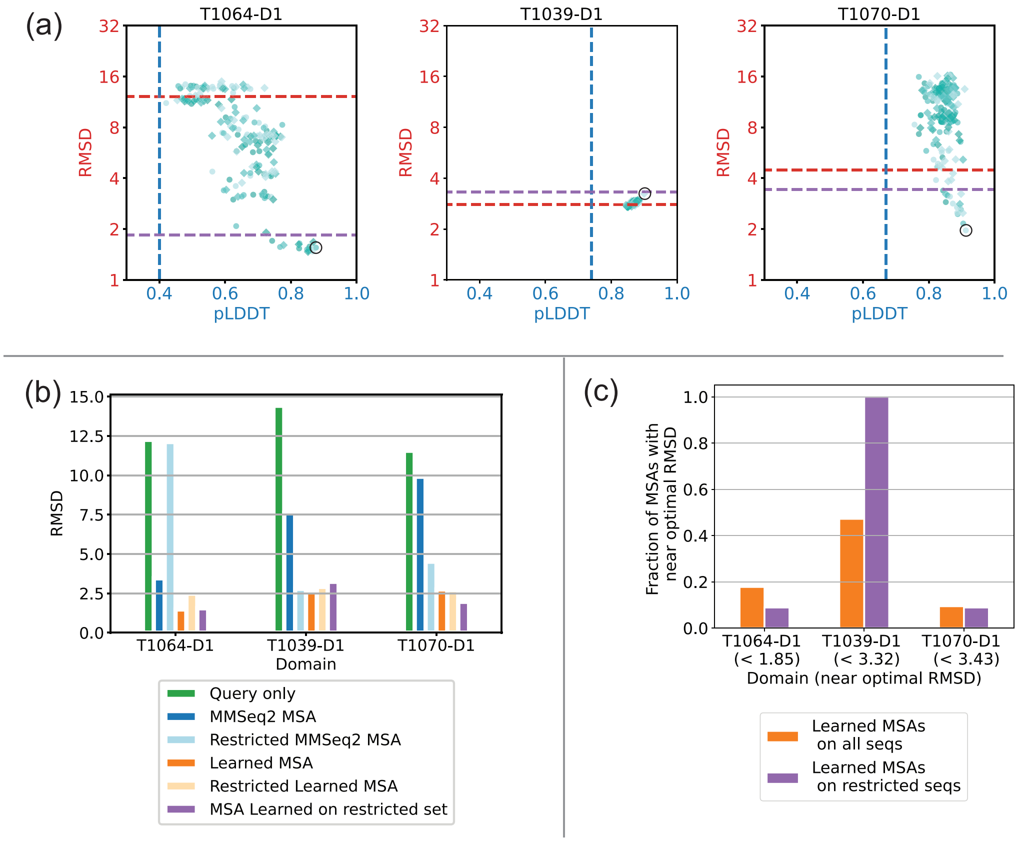

Figure 16: **AlphaFold + LAM optimization on families with distant sequences removed.** (a) Analogous plot to Main Text Figure 3 for LAM + AF experiment with the distant sequences removed. The dotted blue and red lines show the pLDDT and RMSD of the prediction using the MSA from MMseqs2 with the distant sequences removed. The purple line indicates the definition of "near-optimal" and is 1.25 times the RMSD of the prediction for the "Learned MSA" found in Main Text Figures 3 or 4. We selected the circled point maximizing the confidence (pLDDT) as our "MSA Learned on restricted set." (b) A comparison of the RMSD for various tested MSAs, by domain. (c) Fraction of MSAs learned that yielded predictions with "near optimal" structure.

References

Anishchenko, I. *et al.* (2017). Origins of coevolution between residues distant in protein 3d structures. *Proceedings of the National Academy of Sciences*, **114**(34), 9122–9127.

Balakrishnan, S. *et al.* (2011). Learning generative models for protein fold families. *Proteins: Structure, Function, and Bioinformatics*, **79**(4), 1061–1078.

Bepler, T. and Berger, B. (2018). Learning protein sequence embeddings using information from structure. In *International Conference on Learning Representations*.

Bhattacharya, N. *et al.* (2020). Single layers of attention suffice to predict protein contacts. *bioRxiv*.

Dauparas, J. *et al.* (2019). Unified framework for modeling multivariate distributions in biological sequences. *arXiv preprint arXiv:1906.02598*.

Dunn, S. D. *et al.* (2008). Mutual information without the influence of phylogeny or entropy dramatically improves residue contact prediction. *Bioinformatics*, **24**(3), 333–340.

Durbin, R. *et al.* (1998). *Biological sequence analysis: probabilistic models of proteins and nucleic acids*. Cambridge University Press.

Ekeberg, M. *et al.* (2013). Improved contact prediction in proteins: Using pseudolikelihoods to infer Potts models. *Physical Review E - Statistical, Nonlinear, and Soft Matter Physics*, **87**(1).

Gotoh, O. (1982). An improved algorithm for matching biological sequences. *Journal of molecular biology*, **162**(3), 705–708.

Hildebrand, A. *et al.* (2009). Fast and accurate automatic structure prediction with hhpred. *Proteins: Structure, Function, and Bioinformatics*, **77**(S9), 128–132.

Jumper, J. *et al.* (2021a). Applying and improving alphafold at casp14. *Proteins*.

Jumper, J. *et al.* (2021b). Highly accurate protein structure prediction with alphafold. *Nature*, **596**(7873), 583–589.

Kamisetty, H. *et al.* (2013). Assessing the utility of coevolution-based residue–residue contact predictions in a sequence-and structure-rich era. *Proceedings of the National Academy of Sciences*, **110**(39), 15674–15679.

Mensch, A. and Blondel, M. (2018). Differentiable dynamic programming for structured prediction and attention. In *International Conference on Machine Learning*, pages 3462–3471. PMLR.

Mirdita, M. *et al.* (2021). Colabfold-making protein folding accessible to all. *bioRxiv*.

Morton, J. *et al.* (2020). Protein structural alignments from sequence. *BioRxiv*.

Nawrocki, E. P. and Eddy, S. R. (2013). Infernal 1.1: 100-fold faster rna homology searches. *Bioinformatics*, **29**(22), 2933–2935.

Needleman, S. B. and Wunsch, C. D. (1970). A general method applicable to the search for similarities in the amino acid sequence of two proteins. *Journal of molecular biology*, **48**(3), 443–453.

Ovchinnikov, S. *et al.* (2014). Robust and accurate prediction of residue–residue interactions across protein interfaces using evolutionary information. *elife*, **3**, e02030.

Rao, R. *et al.* (2020). Transformer protein language models are unsupervised structure learners. In *International Conference on Learning Representations*.

Smith, T. F. and Waterman, M. S. (1981). Identification of common molecular subsequences. *Journal of molecular biology*, **147**(1), 195–197.

Steinegger, M. *et al.* (2019). HH-suite3 for fast remote homology detection and deep protein annotation. *BMC bioinformatics*, **20**(1), 1–15.

Wozniak, A. (1997). Using video-oriented instructions to speed up sequence comparison. *Bioinformatics*, **13**(2), 145–150.
